# Fluorescence Imaging Using Enzyme-Activatable Probes for Real-Time Identification of Pancreatic Cancer

**DOI:** 10.3389/fonc.2021.714527

**Published:** 2021-08-19

**Authors:** Ryugen Takahashi, Takeaki Ishizawa, Masumitsu Sato, Yoshinori Inagaki, Mariko Takanka, Yugo Kuriki, Mako Kamiya, Tetsuo Ushiku, Yasuteru Urano, Kiyoshi Hasegawa

**Affiliations:** ^1^Hepato-Biliary-Pancreatic Surgery Division, Department of Surgery, Graduate School of Medicine, University of Tokyo, Tokyo, Japan; ^2^Department of Pathology, Graduate School of Medicine, The University of Tokyo, Tokyo, Japan; ^3^Laboratory of Chemistry and Biology, Graduate School of Pharmaceutical Sciences, The University of Tokyo, Tokyo, Japan; ^4^Laboratory of Chemical Biology and Molecular Imaging, Graduate School of Medicine, The University of Tokyo, Tokyo, Japan

**Keywords:** pancreatic cancer, pancreatectomy, fluorescence imaging, activatable probe, intraoperative diagnosis, dipeptidyl peptidase-IV (DPP-IV)

## Abstract

**Introduction:**

Radical resection is the only curative treatment for pancreatic cancer, which is a life-threatening disease. However, it is often not easy to accurately identify the extent of the tumor before and during surgery. Here we describe the development of a novel method to detect pancreatic tumors using a tumor-specific enzyme-activatable fluorescence probe.

**Methods:**

Tumor and non-tumor lysate or small specimen collected from the resected specimen were selected to serve as the most appropriate fluorescence probe to distinguish cancer tissues from noncancerous tissues. The selected probe was sprayed onto the cut surface of the resected specimen of cancer tissue to acquire a fluorescence image. Next, we evaluated the ability of the probe to detect the tumor and calculated the tumor-to-background ratio (TBR) by comparing the fluorescence image with the pathological extent of the tumor. Finally, we searched for a tumor-specific enzyme that optimally activates the selected probe.

**Results:**

Using a library comprising 309 unique fluorescence probes, we selected GP-HMRG as the most appropriate activatable fluorescence probe. We obtained eight fluorescence images of resected specimens, among which four approximated the pathological findings of the tumor, which achieved the highest TBR. Finally, dipeptidyl-peptidase IV (DPP-IV) or a DPP-IV-like enzyme was identified as the target enzyme.

**Conclusion:**

This novel method may enable rapid and real-time visualization of pancreatic cancer through the enzymatic activities of cancer tissues.

## Introduction

Pancreatic cancer is a major life-threatening disease (1–4). Despite recent advances in chemotherapy and radiotherapy, complete resection remains the only curative treatment (5–7). However, it is often difficult to accurately identify the boundaries of cancer tissues during surgery, which may lead to incomplete removal of cancer tissues and unfavorable postoperative survival (6, 7). For patients administered preoperative chemo(radio)therapy (8, 9), it is particularly difficult to identify viable cancer tissues, even in pathological examinations of resected specimens (10).

In 2011, Urano et al. reported a novel fluorescence imaging technique using an “activatable” probe, which is initially nonfluorescent but emits fluorescence immediately after its hydrolysis by γ-glutamyltranspeptidase overexpressed specifically in various cancer cells (11). Subsequently, more than 400 activatable fluorescence probes comprising amino acid or sugar residues that serve as a reactive moiety and target the aminopeptidase or glucosidase have been developed. Furthermore, fluorescently activatable scaffolds such as hydroxymethyl rhodamine-green (HMRG) or hydroxymethyl rhodol with trifluoroethyl group (HMRef) were developed (12), enabling visualization of breast cancer (12–14), esophageal cancer (15, 16), liver cancer (17), lung cancer (18), head and neck cancer (19, 20), colorectal cancer (21), thyroid cancer (22), and glioblastoma (23). Regarding real-time imaging of pancreatic cancer, other approaches using activatable probes can be indicated (24–26), albeit applications of these techniques to fresh human samples have not yet been reported. Here we searched for activatable fluorescence probes for real-time identification of viable pancreatic cancer tissues in resected specimens.

## Material and Methods

The Institutional Review Board of the University of Tokyo Hospital approved this study [IRB No. 2957- (11)].

### Sample Collection

Fresh tissue samples were collected from resected specimens of patients who underwent radical pancreatectomy for pancreatic adenocarcinoma from April 2017 to December 2020. Written informed consent was obtained from all patients. For the primary and secondary probe selection, 3-5 mm-in-size tissue fragments were obtained from obvious cancerous regions and non-cancerous pancreatic parenchyma with confirmation by the pathologist (M.T.), just after the removal of pancreatic specimens.

### Primary Probe Selection

In this study, totally 309 dipeptides-HMRG fluorescence probes were used from our probe library. The concept and synthetic methods of these probes have been described elsewhere (12). Briefly, these probes were synthesized by placing an amino-acid residue selected from 21 amino-acids at P1 and P2 position of Xaa(P2)-Yaa(P1)-HMRG (**Supplementary Table 1**). Among the chemically stable compounds, candidate fluorescence probes were first selected using lysates prepared from cancer and noncancerous tissue samples (12, 15, 23). Briefly, the tissue cut by scissors were homogenized with 1.0 mL of T-per tissue protein in a Lysing Matrix D. After the centrifuge (1,000 rpm x 5 min at 4°C), the supernatant was collected as the lysate. Then, 5 µL of the lysate (0.20 mg/mL protein) was added to the wells of a black 384-well plate, each containing 15 µL of each candidate probe from a library of dipeptide-HMRG compounds (23). The final concentrations of a candidate probe and lysate protein were 1.0 µM and 0.050 mg/dL, respectively. The fluorescence intensity (FI) of each sample was measured using an Envision Multilabel Plate Reader (PerkinElmer, Massachusetts, USA) 0–60 min after the addition of lysates at 37°C. The excitation and emission wavelengths were 485 nm and 535 nm, respectively. The increase of FI was calculated as follows:

(FI increase)=(FI at 60 min)−(FI at 0 min)

Then, we calculated the difference and the ratio of FI increase between cancer and noncancer lysates, and the probes of which difference or ratio represented the ≥ 90th percentile of all probes was subjected to subsequent evaluations.

### Secondary Probe Selection

Candidate fluorescence probes were sprayed directly onto a few millimeters of cancer tissues and noncancerous tissues placed in an eight-well plate. The cancerous and noncancerous tissues were collected as the same way as the primary probe selection, and divided into smaller specimen by scissors respectively. When the size of original tissue samples was insufficient for creating 6 fragments, fluorescence imaging was performed prioritizing candidate probes with better outcomes in the primary screening. The concentration and volume of each fluorescence probe was 50 µM and 200 µL, respectively. Images of fluorescence were obtained using the Maestro *in Vivo* Imaging System (PerkinElmer, Massachusetts, USA), with the blue filter settings (excitation and emission wavelengths of 435–480 nm and 490 nm long pass), respectively, acquired 0 (before), 1, 3, 5, 10, 15, 20, 25, and 30 min after the administration of fluorescence probes. FI was calculated by subtracting the average in the region of interest (ROI) at 1 min from that at 30 min, according to the fluorescence images extracted at 540 nm. Finally, candidate fluorescence probes were refined according to the difference in contrast of FI between cancer tissues and noncancerous tissues.

### Macroscopic Evaluation of Cancer Using Whole Surgical Specimens

Immediately after pancreatic resection, the whole specimen was cut to include the maximum diameter of pancreatic cancer tissues. The selected florescence probe (4 mL, 50 µM solution) was sprayed directly onto the cut surfaces, followed by fluorescence imaging using the Maestro *in Vivo* imaging System, as described above. The accuracy of fluorescence imaging to delineate pancreatic cancer was evaluated by a surgeon (R.T.) and a pathologist (M.T.) with reference to histopathological findings of the same planes. The tumor-to-background ratio (TBR) was calculated as the increase in the mean FI from 1 min to 30 min after administration of the probe to cancer tissues and noncancerous pancreatic tissues, as follows:

$$TBR=\frac{FI\;increase\;of\;the\;cancerous\;tissue}{FI\;increase\;of\;the\;non-cancerous\;tissue}$$

The data were obtained using the Maestro *In Vivo* Imaging System, described above, according to macro- and microscopic pathological findings of the cut surfaces and their corresponding fluorescence images.

### Exploration of Target Enzymes

Through the probe screening process, dipeptidyl peptidases were suspected as target enzymes that may be overexpressed specifically in pancreatic cancer tissues. Thus, we first confirmed the ability of DPP-IV and related enzymes to activate candidate fluorescence probes by measuring changes of FI for 1,000 seconds after the addition of human recombinant DPP-IV (0.040 units; D4943, Sigma-Aldrich), DPP-VIII (1.0 µg; ab162872, abcam), or DPP-IX (1.0 µg; ab79621, abcam) to 3.0 mL of probe (1.0 µM) using the F-7000 Hitachi Fluorescence Spectrophotometer (Hitachi, Tokyo, Japan). The excitation and emission wavelengths were 495 nm and 525 nm, respectively. FI in the cancer tissues used in the secondary screening were also measured after administration of DPP-IV inhibitor (K579, CalbioChem) at a dose of 100 µM. Finally, the expression of DPP-IV on cut surfaces of whole surgical specimens was evaluated using immunohistochemical (IHC) analysis with an anti-DPP-IV mouse monoclonal antibody (TA500733; Origene Technologies Inc, Rockville, MD). Antigen retrieval was performed at 110°C for 15 min. The anti-DPP-IV antibody was diluted 1:100, and the tissues were incubated overnight at 4°C. The IHC results were evaluated by a pathologist (M.T.) uninformed about the outcomes of fluorescence imaging.

## Results

### Primary and Secondary Probe Selection

**Figure 1:**
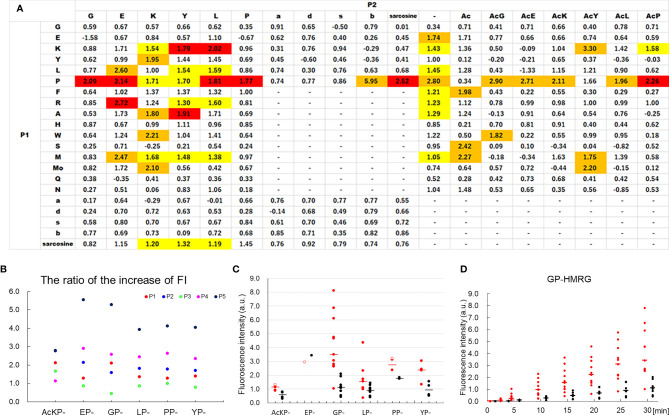
Analysis of FI values of candidate probes in the primary and secondary selections using lysates and tissue fragments obtained from patients with pancreatic cancer. **(A)** The heatmap of the probe library with median differences/ratios of FI increase between cancer and non-cancer lysates for 309 candidate probes. The values ranked in the upper 90 percentile were indicated in yellow (FI differences), orange (FI ratios), or red (both). The number represents the median ratio of FI increase. P1 and P2 correspond the positions of amino acid residues of the probe P2-P1-HMRG. The lowercase letters represent the optical isomers of the amino acids. **(B)** The ratio of the FI increase between cancer and non-cancer lysates obtained from five patients using the selected six HMRG-based fluorescence probes. Probes with a dipeptide with prolyl residue at the P1 position (XaaP-HMRG) tend to show sufficient FI ratios between cancer and non-cancer samples. **(C)** The FI increase 30 min after addition of probe to tissue fragments. The difference in FI increase for 30 minutes tended to be high for GP-HMRG (median [range], 3.49 [1.03–8.11] a.u. *vs* 1.12 [0.42–2.09] a.u., P = 0.002; Wilcoxon’s rank-sum test), followed by AcKP-HMRG (1.14 [0.87–1.29] a.u. *vs* 0.58 [0.29–0.78] a.u., P = 0.008), YP-HMRG (2.39 [1.33–3.04] a.u. *vs* 0.93 [0.52–1.54] a.u., P = 0.043), and LP-HMRG (1.55 [0.35–4.35] a.u. *vs* 0.91 [0.40–1.45] a.u., P = 0.093). The number of the cancer/non-cancer fragments (red filled circle/black filled circle) used in this evaluation was: AcKP- (5/6), EP- (0/1), GP- (11/8), LP-HMRG (7/8), PP- (2/2), and YP-HMRG (4/4). In one case, cancerous samples submitted to fluorescence imaging using EP-, PP-, and AcKP-HMRG were proved to be pancreatitis by final pathological evaluations (red empty circle) and excluded from the statistical analyses. Bars indicate median values. **(D)** Trends of FI increases of cancer tissues and noncancerous tissue fragments obtained from the 11 patients after the administration of GP-HMRG. Bars indicate median values.

Primary selection of fluorescence probes employed lysates prepared from five resected specimens, leading to the identification of candidate probes from 309 fluorescence probes (**Supplementary Table 1**). When the differences and ratios of FI increase between cancer and non-cancer lysates were calculated (**Supplementary Table 2**), 14 out of the 19 HMRG-based fluorescence probes with dipeptides with a prolyl residue at the P1 position (XaaP-HMRG) ranked in the upper 90 percentile (**Figure 1A**). Based not only on the FI differences/ratios but also probe stability and the absolute values of FI increase in non-cancer lysates, which could decrease cancer detectability on tissue samples, AcKP- (Acetylated Lysine-Proline-), EP- (Glutamate-Proline-), GP- (Glycine-Proline-), LP- (Leucine-Proline-), PP (Proline-Proline), and YP-HMRG (Tyrosine-Proline-HMRG) were selected in this study (**Figure 1B**) for the second screening using fresh tissue fragments obtained from 11 patients with pancreatic adenocarcinoma. As a result, fluorescence imaging using GP-HMRG yielded the highest intensity differences in FI after 30 min between cancer and noncancerous tissues [median (range), 3.49 (1.03–8.11) a.u. *vs* 1.12 (0.42–2.09) a.u., P = 0.002; Wilcoxon’s rank-sum test] (**Figures 1C, D**). GP-HMRG was therefore selected to evaluate fluorescence imaging to specifically detect cancer tissues in whole surgical specimens. Demographic background of the totally 16 patients who provided lysates or tissue samples were demonstrated in **Supplementary Table 3**.

### Fluorescence Imaging of Whole Surgical Specimens Using GP-HMRG

Detection of cancer tissues using fluorescence imaging was evaluated by spraying GP-HMRG onto cut surfaces of whole surgical specimens immediately after resection of eight patients with pancreatic adenocarcinoma. Patients’ demographic characteristics are summarized in **Table 1**. Neoadjuvant chemotherapy with gemcitabine and nab-paclitaxel was indicated to two patients who underwent surgery. Three patients were treated for diabetes mellitus but were not preoperatively administered DPP-IV inhibitors.

**Table 1:** Patients’ demographic characteristics and outcomes of fluorescence imaging using GP-HMRG.

Patient	Age (y)	Sex	DM	NAC, (effect*)	Preoperative CA19-9 (IU/mL)	Surgical procedures	Histological type	TBR of FI
1	70	M	−	−	68	DP	Adenosquamous	3.44
2	83	F	+	−	55	DP	tub1 > tub2	2.06
3	63	M	−	−	9	PD	tub2 > tub1 > por	1.98
4	74	M	−	−	39	DP	tub2 > tub1	1.98
5	73	F	+	−	1	DP	tub1 > tub2	1.93
6	68	M	+	−	393	PD	tub2 > tub1	1.47
7	82	F	−	+, (1b*)	25	DP-CAR	tub1 > tub2	1.26
8	84	F	−	+, (1b*)	544	DP	tub1 > tub2**	1.13

DM, diabetic mellitus; NAC, neoadjuvant chemotherapy; PD, pancreaticoduodenectomy; DP, distal pancreatectomy; DP-CAR, distal pancreatectomy with celiac axis resection; TBR, tumor-to-background ratio; tub1/tub2, well/moderately differentiated tubular adenocarcinoma; por, poorly differentiated adenocarcinoma.

*Evans classification.

**With perineural and lymphatic infiltration of viable cancer cells to the splenic artery.

**Figure 2:**
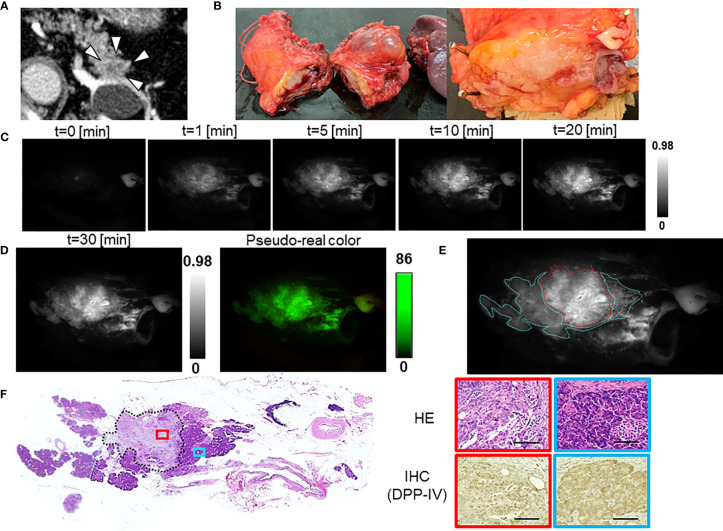
Fluorescence imaging using GP-HMRG to analyze whole surgical specimens demonstrated homogenous increases in fluorescence signals emitted by pancreatic cancer tissues (Patient No. 2). **(A)** Preoperative contrast-enhanced computed tomography of pancreatic tail cancer (arrowheads). **(B)** Macroscopic image of the DP specimen after making cut surfaces of the tumor in the operation room (left). Right indicates magnified view of the cut surface including the tumor. **(C)** Increase of fluorescence signals after spraying GP-HMRG on the cut surface. **(D)** Fluorescence image (left) and its pseudo-real color image (right) of the cut surface 30 min after probe administration. **(E)** Relationships between fluorescence signals and distributions of cancer tissues (red dotted line) and surrounding pancreatic tissues (blue dotted line) according to histological findings. **(F)** Low-magnification histopathological image of hematoxylin–eosin (H&E) staining corresponding to fluorescence images (left, dotted line indicates cancer boundaries). Magnified views of H&E staining and IHC analysis of DPP-IV in cancer (red) and pancreatic (blue) tissues (right). Scale bar = 100 µm.

**Figure 3:**
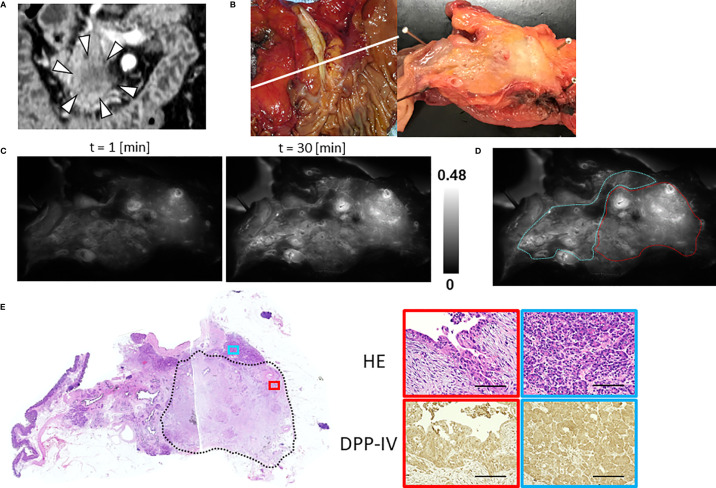
Fluorescence imaging using GP-HMRG to probe whole surgical specimens demonstrates heterogenous increases in fluorescence signals emitted by pancreatic cancer tissues (Patient No. 6). **(A)** Preoperative contrast-enhanced computed tomography of pancreatic head cancer (arrowheads). **(B)** Macroscopic image of the PD specimen (left) and cut surface along the white line including the tumor (right). **(C)** Increase of fluorescence signals after spraying GP-HMRG on the cut surface. **(D)** Relationships between fluorescence signals and distributions of cancer tissues (red dotted line) and surrounding pancreatic tissues (blue dotted line) according to histological findings. **(E)** Low-magnification histopathological image of H&E staining corresponding to fluorescence images (left, dotted line indicates cancer boundaries). Magnified views of H&E staining and IHC analysis of DPP-IV in cancer (red) and pancreatic (blue) tissues (right). Scale bar = 100 µm.

**Figure 4:**
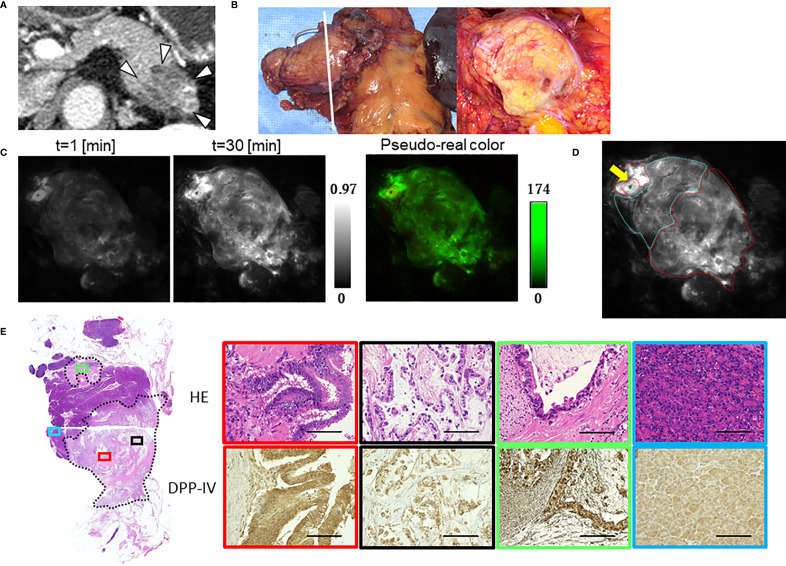
Fluorescence imaging using GP-HMRG to probe whole surgical specimens demonstrates cancer infiltration to the splenic artery (Patient No. 8). **(A)** Preoperative contrast-enhanced computed tomography of pancreatic body cancer (arrowheads). **(B)** Macroscopic images of the DP specimen (left) and cut surface along the dotted line including the tumor (right). **(C)** Increase of fluorescence signals after spraying GP-HMRG on the cut surface and pseudo-real color image at 30 min. **(D)** Relationships between fluorescence signals and distributions of cancer tissues (red dotted line) and surrounding pancreatic tissues (blue dotted line) according to histological findings. Arrow indicates the splenic artery. **(E)** Low-magnification histopathological image of H&E staining corresponding to fluorescence images (left, dotted line indicates cancer boundaries). Magnified views of H&E staining and IHC analysis of DPP-IV showing fluorescence (red) and little fluorescence (black) emitted by parts of the main tumor, viable cancer infiltration around the splenic artery (green), and noncancerous pancreatic tissues (blue), (right). Scale bar = 100 µm.

The median TBR of the fluorescence images following the administration of GP-HMRG was 1.96 (range, 1.13–3.44). In five patients with TBRs ranging from 1.93 to 3.44, fluorescence signals in cancer tissues were nearly homogenous and grossly discriminable from the surrounding pancreatic tissues (**Figure 2**). In the remaining three patients, including two who underwent preoperative chemotherapy, cancer tissues emitted heterogenous fluorescence signals, making it difficult to discriminate them from noncancer tissues (**Figure 3**). Fluorescence imaging identified a significant signal increase (TBR, 2.04) in the connective tissues surrounding the splenic artery of one patient in the latter group, which pathological examination subsequently diagnosed as perineural and lymphatic infiltration by viable cancer cells, while the main tumor included fibrosis and mucinous changes with a few viable cancer cells (TBR, 1.13), likely caused by preoperative chemotherapy (**Figure 4**).

### Identification of Target Enzymes That Activate GP-HMRG

**Figure 5:**
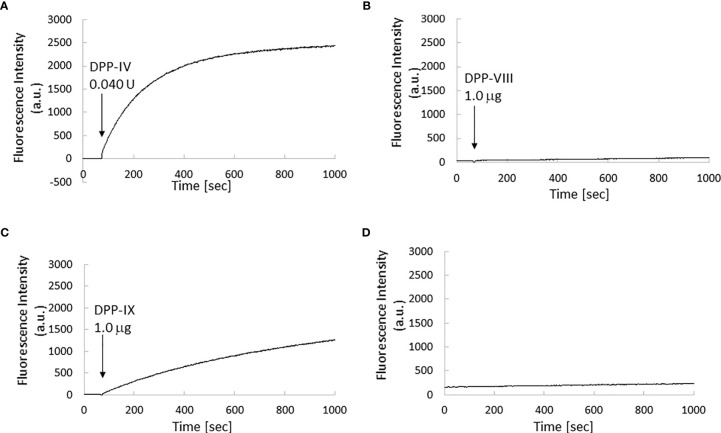
Kinetics of fluorescence intensity of GP-HMRG upon enzyme addition. µ Excitation/emission wavelengths were 495 nm/525 nm. Trends of fluorescence intensities after adding DPP-IV (0.040 units, 0.013 units/ml; **(A)**, DPP-VIII (1.0 µg, 0.033 µg/ml; **(B)**, or DPP-IX (1.0 µg, 0.033 µg/ml; **(C)** to GP-HMRG. Increase of fluorescence intensity of GP-HMRG with no addition of enzymes was also demonstrated in panel **(D)**.

**Figure 6:**
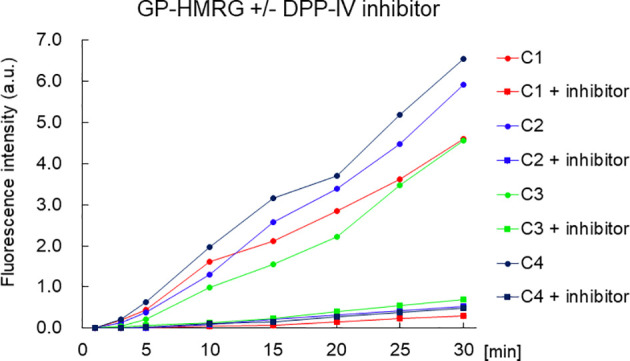
The time course of the increase of FI of small cancer specimen with or without inhibitor. The fluorescence intensity of the pancreatic cancer specimen was decreased with DPP-IV inhibitor. The concentration of the DPP-IV inhibitor (K579, CalbioChem) was 100 µM.

The *in vitro* fluorescence spectrum of GP-HMRG after adding DPPs indicated that the probe was converted to highly fluorescent HMRG upon reaction with DPP-IV and DPP-IX (**Figure 5**). On the cancer tissue specimens used in the secondary screening (available in 4 cases), FI increase was markedly suppressed when GP-HMRG was administered with the inhibitor (**Figure 6**). In contrast, IHC analysis of the resected specimens of eight patients did not detect an unambiguous difference in the expression levels of DPP-IV between cancer and surrounding pancreatic tissues (**Figures 2**–**4**).

## Discussion

Here we screened GP-HMRG, among 309 candidates of activatable fluorescence probes, for its ability to specifically identify pancreatic cancer tissues. Fluorescence imaging using GP-HMRG sprayed onto cut surfaces of fresh resected specimens visualized cancer tissues as homogenous fluorescing regions with a high (>1.9) TBR in five of eight patients. Among the remaining three patients, fluorescence signals in cancer tissues were heterogenous and therefore insufficient for unambiguous discrimination from surrounding noncancerous tissues. However, in one patient who underwent preoperative chemotherapy, fluorescence imaging visualized grossly-unidentifiable cancer infiltrations around the splenic artery. These results suggest that fluorescence imaging using GP-HMRG potentially visualizes the spread of pancreatic cancer cells in real time, which may be useful for intraoperative diagnosis of surgical margins as well as for preoperative endoscopic evaluations of intraductal lesions.

The major advantage of using activatable probes is their ability to rapidly identify in real-time cancer tissues according to enzymatic activities, which are specifically expressed by cancer cells. Moreover, in the present series, an increase in fluorescence signals emitted by cancer tissues was identified 1 min after the topical administration of GP-HMRG. The FI values of cancer tissues in the remaining three patients may be decreased because of fewer viable cancer cells with fibrosis and mucinous changes, likely caused by preoperative chemotherapy.

Recently developed fluorescence imaging techniques for intraoperative identification of pancreatic cancer employ 5-aminolevulinic acid (27), indocyanine green (28), novel fluorophores targeting carbohydrate antigen 19-9 (CA19-9) (29), carcinoembryonic antigen (CEA) (30–35), epidermal growth factor receptor (EGFR) (36, 37), and insulin-like growth factor 1 receptor (IGF-1R) (38). However, these techniques, most of which involve systemic administration of “non-activatable” probes, usually require longer intervals for washout of fluorescence agents from background tissues, which may lead to lower TBRs compared with those of activatable probes topically administered during surgery.

The present technique offers potential advantages for detecting tumor cells with high enzymatic activities specific to cancer tissues, which may facilitate prediction of a patient’s sensitivity to chemotherapy and postoperative outcome, as previously suggested in case of fluorescence imaging of colorectal liver metastasis by gGlu-HMRG (17).

The most likely candidate as a target enzyme of GP-HMRG is DPP-IV that cleaves the N-terminal residue of Xaa-Pro/Ala to regulate the bioavailability of glucose-insulinotropic peptide (GIP) and glucagon-like peptide-1 (GLP-1) (39). Other studies demonstrate the upregulation of DPP-IV in malignancies (40–42) such as pancreatic cancer (43), although IHC analysis here did not demonstrate unambiguous differences in the expression levels of DPP-IV between cancer tissues and noncancerous tissues, likely because of the different antibodies used to detect DPP-IV or for other technical reasons. Another candidate enzyme is DPP-IX, which is upregulated in pancreatic cancer tissues (43) and cleaves GP-HMRG, although IHC staining of DPP-IX expression sufficient for pathological analysis was unavailable.

The limitation of this study lies in its small sample size. With the sufficient number of lysates and tissue samples for robust statistical analyses, more promising fluorophores other than GP-HMRG might have been identified in the initial screening processes. Considering the heterogeneity of pancreatic cancers and factors affecting the enzymatic activities of cancer tissues, we must continue to conduct evaluation of the efficiency of GP-HMRG for fluorescence imaging to identify cancer tissue using an observer-blinded trial, as well as to predict postoperative outcomes of a larger population. Furthermore, the potentially insufficient levels of DPP-IV in cancer cells, as well as in stromal and Langerhans islets (43, 44), may decrease the sensitivity of cancer detection in certain patients. The expression status of DPP-IV and DPP-IX must therefore be evaluated using IHC and the diced electrophoresis gel assay (DEG-Assay) (45).

In conclusion, fluorescence imaging using GP-HMRG may enable rapid and real-time visualization of pancreatic cancer through the detection of cancer tissue-specific enzymatic activities.

## Data Availability Statement

The raw data supporting the conclusions of this article will be made available by the authors, without undue reservation.

## Ethics Statement

The studies involving human participants were reviewed and approved by The Institutional Review Board of the University of Tokyo Hospital (IRB No. 2957-[11]). The patients/participants provided their written informed consent to participate in this study. Written informed consent was obtained from the individual(s) for the publication of any potentially identifiable images or data included in this article.

## Author Contributions

RT, MS, and TI drafted the initial manuscript. RT and MS collected the samples, acquired the data, and performed the analysis. YK and MK prepared a library of activatable fluorescence probes. RT and MT evaluated the pathological extent of tumor. RT and YI performed immunohistochemistry, and MT evaluated the results. MK, TU, YU, and KH critically evaluated and revised the manuscript. All authors contributed to the article and approved the submitted version.

## Funding

This work was supported by collaborative research funding between The University of Tokyo and NIPRO Corporation, and grants from the Ministry of Education, Culture, Sports, Science and Technology of Japan [Grant Number 19H05632 to YU]. The funder was not involved in the study design, collection, analysis, interpretation of data, the writing of this article or the decision to submit it for publication.

## Conflict of Interest

The authors declare that the research was conducted in the absence of any commercial or financial relationships that could be construed as a potential conflict of interest.

## Publisher’s Note

All claims expressed in this article are solely those of the authors and do not necessarily represent those of their affiliated organizations, or those of the publisher, the editors and the reviewers. Any product that may be evaluated in this article, or claim that may be made by its manufacturer, is not guaranteed or endorsed by the publisher.
