# Supplementary material for: Fluorescence Imaging Using Enzyme-Activatable Probes for Real-Time Identification of Pancreatic Cancer
Source: Front Oncol. 2021 Aug 19;11:714527. doi: 10.3389/fonc.2021.714527 (PMC8417470; doi:10.3389/fonc.2021.714527)

Supplementary Material

# Supplementary Table

## Supplementary Table 1. FI values of all probes in the primary selection set using lysates obtained from patients with pancreatic cancer. P1 and P2 correspond the positions of amino acid residues of the probe P2-P1-HMRG. The lowercase letters represent the optical isomers of the amino acids.

|  |  | **P2** |  |  |  |  |  |  |  |  |  |  |  |  |  |  |  |  |  |  |
| --- | --- | --- | --- | --- | --- | --- | --- | --- | --- | --- | --- | --- | --- | --- | --- | --- | --- | --- | --- | --- |
|  |  | **G** | **E** | **K** | **Y** | **L** | **P** | **a** | **d** | **s** | **b** | **sarcosine** | **-** | **Ac** | **AcG** | **AcE** | **AcK** | **AcY** | **AcL** | **AcP** |
| **P1** | **G** | **656963** | **-400524** | **107658** | **484882** | **185769** | **-724170** | **-10146** | **-945874** | **-993744** | **-527369** | **-603889** | **-402111** | **-260288** | **-921044** | **-1244442** | **-1125285** | **-754700** | **-987132** | **-2203777** |
|  | **E** | **-2702979** | **-1672976** | **-1324812** | **-1255468** | **194599** | **-2223944** | **-2039615** | **-1935544** | **-1833370** | **-119962** | **-909808** | **967885** | **1122312** | **-2436465** | **-1934137** | **-1461475** | **-1907427** | **-2555556** | **-2182944** |
|  | **K** | **16977055** | **-1079985** | **3391970** | **12818752** | **8228851** | **25015555** | **96060** | **-2493473** | **2017266** | **-1548251** | **1822985** | **19012753** | **147132** | **-1012446** | **-748153** | **18145407** | **-607931** | **-234458** | **6029702** |
|  | **Y** | **5821752** | **74773** | **981867** | **2677931** | **1536051** | **4829399** | **520** | **-273805** | **-133250** | **-257013** | **-136209** | **105868** | **-634804** | **-400979** | **-322289** | **-523930** | **-265395** | **-441229** | **-577576** |
|  | **L** | **3105880** | **3385229** | **19290** | **5863942** | **8490317** | **5334866** | **-352039** | **-282079** | **-193810** | **-481336** | **-483446** | **20577182** | **43368** | **-91815** | **-143175** | **102604** | **6863** | **26544** | **82144** |
|  | **P** | **13486668** | **12486653** | **31895712** | **11240924** | **15638367** | **22342891** | **2195384** | **6122793** | **13912503** | **-10181** | **4200555** | **314386** | **-483777** | **414203** | **261658** | **3920279** | **2073215** | **2881327** | **4339537** |
|  | **F** | **3722348** | **452879** | **405020** | **1757027** | **1807799** | **3329248** | **-** | **-** | **-** | **-** | **-** | **4037254** | **846390** | **-514983** | **-473418** | **-645320** | **-158622** | **-214300** | **-297218** |
|  | **R** | **30063161** | **2220107** | **5336865** | **13185036** | **10780273** | **24189713** | **-** | **-** | **-** | **-** | **-** | **27947289** | **422015** | **6177901** | **3190029** | **44332067** | **4777836** | **6678525** | **27495514** |
|  | **A** | **4572085** | **-824633** | **6656512** | **6363446** | **5597054** | **2613953** | **-** | **-** | **-** | **-** | **-** | **24889068** | **137078** | **-823889** | **-702216** | **-1037016** | **-384606** | **-655641** | **-1224595** |
|  | **H** | **4414474** | **45477** | **149494** | **791578** | **553916** | **3893412** | **-** | **-** | **-** | **-** | **-** | **273074** | **-195131** | **-906699** | **-250582** | **-173171** | **-107739** | **-63428** | **-413694** |
|  | **W** | **5693720** | **-30214** | **680135** | **679940** | **743395** | **2158771** | **-** | **-** | **-** | **-** | **-** | **2585718** | **-39191** | **-29248** | **-19772** | **-392714** | **52516** | **26144** | **-14801** |
|  | **S** | **387692** | **-3373794** | **-2160861** | **-1952955** | **-1168603** | **-686887** | **-** | **-** | **-** | **-** | **-** | **1014586** | **1988691** | **-760341** | **-1503780** | **-835565** | **-522586** | **-973196** | **-1679564** |
|  | **M** | **14764312** | **1115950** | **8346478** | **7649605** | **7350378** | **12059364** | **-** | **-** | **-** | **-** | **-** | **33671746** | **497607** | **-815862** | **-428141** | **-126845** | **89120** | **131766** | **-15627** |
|  | **Mo** | **4441930** | **-76969** | **635953** | **145981** | **175410** | **3616223** | **-** | **-** | **-** | **-** | **-** | **3102469** | **-73679** | **-202774** | **-297505** | **-168704** | **-36274** | **-1551254** | **-210483** |
|  | **Q** | **5384914** | **-2892510** | **-3004058** | **-1279345** | **-2087010** | **3104467** | **-** | **-** | **-** | **-** | **-** | **4324037** | **-895024** | **-1396482** | **-1507874** | **-1243685** | **-1590632** | **-1520458** | **-2160302** |
|  | **N** | **409580** | **-2657879** | **-2696066** | **-1666175** | **-554046** | **-106463** | **-** | **-** | **-** | **-** | **-** | **844167** | **-2678** | **-625400** | **-892010** | **-599709** | **-972672** | **-97832** | **-765454** |
|  | **a** | **-311064** | **-1551336** | **-821453** | **-1534702** | **-1398574** | **-1802950** | **-780701** | **-679749** | **-149229** | **-501385** | **-479122** | **-** | **-** | **-** | **-** | **-** | **-** | **-** | **-** |
|  | **d** | **57010** | **-2470004** | **-2989064** | **-1523445** | **-1357376** | **-2459479** | **-1122725** | **-2644859** | **-132941** | **-322090** | **-1064422** | **-** | **-** | **-** | **-** | **-** | **-** | **-** | **-** |
|  | **s** | **-726638** | **-1627100** | **-1263578** | **-1277758** | **-1341853** | **-1404166** | **-1106534** | **-1011362** | **-73574** | **-1338216** | **-1401240** | **-** | **-** | **-** | **-** | **-** | **-** | **-** | **-** |
|  | **b** | **-439689** | **-487143** | **-378473** | **-105824** | **-290434** | **-320486** | **-536021** | **-424929** | **-226993** | **-442693** | **-362960** | **-** | **-** | **-** | **-** | **-** | **-** | **-** | **-** |
|  | **sarcosine** | **6814400** | **7939272** | **26703288** | **15217340** | **16037554** | **6267207** | **12531694** | **11827390** | **28032599** | **-863831** | **4059360** | **-** | **-** | **-** | **-** | **-** | **-** | **-** | **-** |

Cancer 1

Normal 1

|  |  | **P2** |  |  |  |  |  |  |  |  |  |  |  |  |  |  |  |  |  |  |
| --- | --- | --- | --- | --- | --- | --- | --- | --- | --- | --- | --- | --- | --- | --- | --- | --- | --- | --- | --- | --- |
|  |  | **G** | **E** | **K** | **Y** | **L** | **P** | **a** | **d** | **s** | **b** | **sarcosine** | **-** | **Ac** | **AcG** | **AcE** | **AcK** | **AcY** | **AcL** | **AcP** |
| **P1** | **G** | **98996** | **-597973** | **-45243** | **215026** | **-419576** | **-2040823** | **-89963** | **-1306861** | **-1373257** | **-724611** | **-860257** | **-1724394** | **-367094** | **-1421169** | **-1752337** | **-1696349** | **-1275057** | **-1260404** | **-3124293** |
|  | **E** | **-3210237** | **-2506738** | **-1476254** | **-2189451** | **-798234** | **-3630261** | **-2703587** | **-2528206** | **-2807326** | **-154652** | **-1298413** | **615949** | **657820** | **-3183265** | **-2946804** | **-2206796** | **-3040547** | **-4108270** | **-3459857** |
|  | **K** | **4139132** | **-3269636** | **-284472** | **7141520** | **4073181** | **15578622** | **-2267070** | **-3274364** | **1260071** | **-2151788** | **-332716** | **14320453** | **108044** | **-2009176** | **-904977** | **17457514** | **-3375839** | **158459** | **5101701** |
|  | **Y** | **3417191** | **-170163** | **503425** | **1729507** | **945877** | **2388646** | **-202686** | **-378238** | **-433971** | **-354215** | **-414785** | **106191** | **-1022723** | **-596436** | **-552211** | **-805551** | **-417578** | **-735800** | **-786911** |
|  | **L** | **1426435** | **2444947** | **14112** | **4026415** | **5352223** | **1139385** | **-431899** | **-401461** | **-400662** | **-766401** | **-466356** | **12916427** | **-48906** | **-214940** | **-179574** | **-19085** | **-20459** | **-53435** | **-98370** |
|  | **P** | **6452312** | **9864204** | **22909798** | **8065984** | **11676486** | **17505360** | **1235606** | **3456125** | **7978998** | **-50972** | **3295623** | **74945** | **-960961** | **30356** | **-28429** | **1856575** | **1249803** | **1471242** | **2038101** |
|  | **F** | **1284389** | **-437646** | **-241638** | **431663** | **311193** | **1175992** | **-** | **-** | **-** | **-** | **-** | **612148** | **427309** | **-937941** | **-817963** | **-1170566** | **-525063** | **-991280** | **-1092550** |
|  | **R** | **18936210** | **1372780** | **4315360** | **10109776** | **7667006** | **13036309** | **-** | **-** | **-** | **-** | **-** | **19211582** | **50409** | **7893734** | **4102104** | **45426454** | **4911492** | **6763203** | **27455419** |
|  | **A** | **1076694** | **-2130546** | **3693300** | **3329405** | **3264186** | **478775** | **-** | **-** | **-** | **-** | **-** | **14166064** | **-2588970** | **-1508293** | **-767822** | **-1876396** | **-710666** | **-1413021** | **-2214673** |
|  | **H** | **2780387** | **-137578** | **-164991** | **342775** | **-68401** | **2174601** | **-** | **-** | **-** | **-** | **-** | **-108567** | **-323573** | **-1301350** | **-310452** | **-117792** | **-89971** | **-143402** | **-664014** |
|  | **W** | **2953167** | **-194618** | **307425** | **-200286** | **316180** | **809612** | **-** | **-** | **-** | **-** | **-** | **1082742** | **-78449** | **-53837** | **-60465** | **-711822** | **24846** | **-15685** | **-34945** |
|  | **S** | **-1766071** | **-5057847** | **-3370746** | **-3139484** | **-3373692** | **-2835926** | **-** | **-** | **-** | **-** | **-** | **335343** | **794731** | **-2030159** | **-2532899** | **-1735662** | **-1174372** | **-2374600** | **-3236520** |
|  | **M** | **8992454** | **448145** | **4981255** | **3936627** | **3251202** | **6431608** | **-** | **-** | **-** | **-** | **-** | **20721006** | **143425** | **-1500825** | **-366548** | **-247962** | **10378** | **-80858** | **-140166** |
|  | **Mo** | **2540680** | **-136299** | **261001** | **-423481** | **-444496** | **2170772** | **-** | **-** | **-** | **-** | **-** | **1552889** | **-193992** | **-357532** | **-410813** | **-193528** | **-96654** | **-2713472** | **-297183** |
|  | **Q** | **-247708** | **-4808836** | **-2799817** | **-3497867** | **-6355868** | **-2118337** | **-** | **-** | **-** | **-** | **-** | **2406705** | **-1380864** | **-2096856** | **-2066325** | **-1833896** | **-2318106** | **-2092893** | **-2628119** |
|  | **N** | **-2317048** | **-4393724** | **-4596776** | **-1384292** | **-1500045** | **-974193** | **-** | **-** | **-** | **-** | **-** | **333100** | **-265297** | **-1180822** | **-1364052** | **-1142328** | **-1257288** | **-424574** | **-1360856** |
|  | **a** | **-749422** | **-2194805** | **-1296199** | **-2302538** | **-2168908** | **-2732503** | **-1023889** | **-835993** | **-193516** | **-650713** | **-582721** | **-** | **-** | **-** | **-** | **-** | **-** | **-** | **-** |
|  | **d** | **-352378** | **-3441870** | **-4179245** | **-1843701** | **-1722853** | **-3274017** | **-1713798** | **-4111102** | **-201637** | **-405619** | **-1615602** | **-** | **-** | **-** | **-** | **-** | **-** | **-** | **-** |
|  | **s** | **-1242428** | **-2028214** | **-1807162** | **-1896745** | **-1989004** | **-708993** | **-1577395** | **-1441199** | **-134756** | **-1939362** | **-1586639** | **-** | **-** | **-** | **-** | **-** | **-** | **-** | **-** |
|  | **b** | **-588676** | **-704720** | **-515447** | **-128831** | **-401988** | **-471289** | **-629510** | **-513236** | **-281928** | **-540680** | **-211808** | **-** | **-** | **-** | **-** | **-** | **-** | **-** | **-** |
|  | **sarcosine** | **4760304** | **4079730** | **16437472** | **9598496** | **10686164** | **4319281** | **7371107** | **5192168** | **16139578** | **-1170055** | **2436989** | **-** | **-** | **-** | **-** | **-** | **-** | **-** | **-** |

Cancer 2

|  |  | **P2** |  |  |  |  |  |  |  |  |  |  |  |  |  |  |  |  |  |  |
| --- | --- | --- | --- | --- | --- | --- | --- | --- | --- | --- | --- | --- | --- | --- | --- | --- | --- | --- | --- | --- |
|  |  | **G** | **E** | **K** | **Y** | **L** | **P** | **a** | **d** | **s** | **b** | **sarcosine** | **-** | **Ac** | **AcG** | **AcE** | **AcK** | **AcY** | **AcL** | **AcP** |
| **P1** | **G** | **5817349** | **1703866** | **8446632** | **9022435** | **8576979** | **9398478** | **396694** | **-60313** | **719321** | **-22491** | **318960** | **7443712** | **-96010** | **420971** | **-469127** | **-67064** | **39519** | **197148** | **-916944** |
|  | **E** | **3504131** | **-28370** | **2209882** | **1834165** | **5525099** | **1970643** | **-425258** | **-527974** | **-144621** | **-31868** | **-130604** | **4688818** | **7003036** | **-949903** | **-800086** | **-644313** | **-753328** | **-826026** | **-897601** |
|  | **K** | **59840380** | **17100736** | **84380138** | **89515310** | **78980465** | **87549027** | **13047831** | **-228251** | **12912626** | **1123184** | **24199616** | **88783765** | **485252** | **4117011** | **2140610** | **25422566** | **5309425** | **6349066** | **14000286** |
|  | **Y** | **40220474** | **15240045** | **38388643** | **32883046** | **30933435** | **42056134** | **1944778** | **187525** | **3296493** | **323535** | **1752930** | **9519468** | **962864** | **408191** | **129673** | **186409** | **363841** | **247912** | **16685** |
|  | **L** | **30664585** | **40421467** | **67756** | **49583056** | **40048815** | **50981472** | **1387669** | **250346** | **2550102** | **2120134** | **580673** | **86771054** | **988592** | **462784** | **148056** | **1003742** | **333569** | **474199** | **228108** |
|  | **P** | **58483877** | **54823687** | **81397836** | **38820780** | **54894483** | **86852034** | **2133705** | **6204370** | **11112453** | **883069** | **26307372** | **2545084** | **-143535** | **4668846** | **2055105** | **21569114** | **13549760** | **14006683** | **19614312** |
|  | **F** | **20170253** | **18106982** | **24016274** | **24915811** | **14626186** | **23082362** | **-** | **-** | **-** | **-** | **-** | **30897125** | **3733345** | **922610** | **318295** | **286678** | **342091** | **974874** | **259983** |
|  | **R** | **99603673** | **37862882** | **68533753** | **99478203** | **80986636** | **75314511** | **-** | **-** | **-** | **-** | **-** | **91145843** | **2513876** | **9218663** | **5450455** | **46369732** | **10701250** | **12358581** | **36343832** |
|  | **A** | **48426910** | **26658297** | **90895654** | **71094287** | **75107520** | **30786306** | **-** | **-** | **-** | **-** | **-** | **95664952** | **13633586** | **616215** | **259742** | **1478538** | **997740** | **1668168** | **985044** |
|  | **H** | **23703464** | **782576** | **4254296** | **5195197** | **5352098** | **26336717** | **-** | **-** | **-** | **-** | **-** | **4105042** | **143643** | **-224922** | **-118094** | **-25192** | **130442** | **319098** | **-188587** |
|  | **W** | **20397115** | **4139643** | **17867094** | **9285205** | **8579448** | **14556722** | **-** | **-** | **-** | **-** | **-** | **18157851** | **42007** | **151531** | **42043** | **112832** | **157433** | **181753** | **31381** |
|  | **S** | **12030115** | **106112** | **6484964** | **7578647** | **7419414** | **10405536** | **-** | **-** | **-** | **-** | **-** | **10727324** | **13671607** | **1810135** | **-239378** | **569211** | **1085923** | **1095705** | **-306832** |
|  | **M** | **73879217** | **25597098** | **77115602** | **55432736** | **57131886** | **64991901** | **-** | **-** | **-** | **-** | **-** | **108692567** | **3972008** | **1943892** | **188672** | **739110** | **894243** | **774369** | **114810** |
|  | **Mo** | **34135182** | **2217512** | **17382913** | **17425887** | **17836218** | **30249645** | **-** | **-** | **-** | **-** | **-** | **26215493** | **658284** | **-46460** | **-137005** | **67045** | **332174** | **293683** | **164867** |
|  | **Q** | **44567594** | **2353496** | **20418455** | **31013115** | **29683540** | **45692248** | **-** | **-** | **-** | **-** | **-** | **37236563** | **478061** | **-82902** | **-691457** | **-228856** | **-147805** | **411063** | **-631682** |
|  | **N** | **16564596** | **9732** | **2643901** | **5403251** | **9978234** | **8689315** | **-** | **-** | **-** | **-** | **-** | **4707478** | **2363949** | **6096** | **-497212** | **-162117** | **-246378** | **223332** | **-371278** |
|  | **a** | **175226** | **-446267** | **241652** | **-172178** | **20374** | **-672898** | **-216347** | **-192063** | **64003** | **-153007** | **-133064** | **-** | **-** | **-** | **-** | **-** | **-** | **-** | **-** |
|  | **d** | **506058** | **-413473** | **-1082713** | **-377944** | **-81246** | **-1169375** | **911** | **-800017** | **-15908** | **-99164** | **-313113** | **-** | **-** | **-** | **-** | **-** | **-** | **-** | **-** |
|  | **s** | **-125925** | **-450917** | **-196296** | **-225272** | **-288038** | **-441717** | **-238507** | **-500231** | **-34678** | **-692296** | **-18720** | **-** | **-** | **-** | **-** | **-** | **-** | **-** | **-** |
|  | **b** | **-171477** | **-241924** | **151434** | **111401** | **47137** | **-138117** | **-166701** | **-157294** | **-95124** | **-143330** | **-149979** | **-** | **-** | **-** | **-** | **-** | **-** | **-** | **-** |
|  | **sarcosine** | **7962877** | **8678473** | **39894722** | **28183597** | **32090361** | **18153497** | **9739191** | **10661765** | **25360765** | **-397164** | **3709583** | **-** | **-** | **-** | **-** | **-** | **-** | **-** | **-** |

Normal 2

|  |  | **P2** |  |  |  |  |  |  |  |  |  |  |  |  |  |  |  |  |  |  |
| --- | --- | --- | --- | --- | --- | --- | --- | --- | --- | --- | --- | --- | --- | --- | --- | --- | --- | --- | --- | --- |
|  |  | **G** | **E** | **K** | **Y** | **L** | **P** | **a** | **d** | **s** | **b** | **sarcosine** | **-** | **Ac** | **AcG** | **AcE** | **AcK** | **AcY** | **AcL** | **AcP** |
| **P1** | **G** | **4855749** | **-126907** | **1442329** | **3722050** | **3910973** | **5548834** | **248089** | **-1154033** | **-394694** | **-668846** | **26697651** | **2828295** | **-423241** | **-1220683** | **-1780367** | **-1435368** | **-1033397** | **-688832** | **-3298997** |
|  | **E** | **-893209** | **-1982081** | **-1110345** | **-845405** | **1406225** | **-652573** | **-2678617** | **-2467386** | **-2203841** | **-122957** | **-1402002** | **2701989** | **2121649** | **-3198642** | **-2895552** | **-2387709** | **-3258305** | **-3340294** | **-3382063** |
|  | **K** | **67996690** | **5699307** | **28746545** | **47561433** | **26931965** | **88739068** | **10984703** | **-3003361** | **12398650** | **-1285641** | **21811606** | **55117769** | **478655** | **1482919** | **-293654** | **25920597** | **1606504** | **4481763** | **8871109** |
|  | **Y** | **29248590** | **2878587** | **9171326** | **14974125** | **10950184** | **29139387** | **1379021** | **-311661** | **2351779** | **-83683** | **1080370** | **3568292** | **-863746** | **-524098** | **-622056** | **-649851** | **257861** | **-689087** | **-563153** |
|  | **L** | **20555771** | **13818080** | **142115** | **25237213** | **30098489** | **31507693** | **705723** | **-339838** | **1600733** | **856513** | **112721** | **62145308** | **151879** | **-80948** | **-111512** | **607293** | **220695** | **367048** | **17219** |
|  | **P** | **37175257** | **25653006** | **47544281** | **22869997** | **30311563** | **48959253** | **2901664** | **8029999** | **13190176** | **65550** | **10436180** | **639051** | **-1383880** | **661139** | **288754** | **7830434** | **4257715** | **5527426** | **8137575** |
|  | **F** | **20644588** | **2347385** | **3448054** | **10123080** | **5349559** | **17797518** | **-** | **-** | **-** | **-** | **-** | **15699430** | **1112745** | **-1000148** | **-1230656** | **-1474548** | **-435713** | **-503318** | **-1186384** |
|  | **R** | **117677482** | **13914227** | **28186366** | **56183610** | **44697168** | **92739731** | **-** | **-** | **-** | **-** | **-** | **74215597** | **2941280** | **13113587** | **5495143** | **52291052** | **10700321** | **13166206** | **40398336** |
|  | **A** | **30175631** | **5799664** | **33287032** | **34462292** | **28266505** | **21673650** | **-** | **-** | **-** | **-** | **-** | **69260368** | **1555901** | **-815975** | **-1220825** | **79425** | **-310991** | **-468911** | **-2350820** |
|  | **H** | **27230567** | **67072** | **1023382** | **2562552** | **3242618** | **25873774** | **-** | **-** | **-** | **-** | **-** | **1631479** | **-261849** | **-1218919** | **-457855** | **-256735** | **-31123** | **217353** | **-822221** |
|  | **W** | **19175054** | **659022** | **3797412** | **3709025** | **3279194** | **14913271** | **-** | **-** | **-** | **-** | **-** | **8514869** | **-20434** | **31732** | **-46000** | **-466295** | **183347** | **181504** | **-21462** |
|  | **S** | **9351175** | **-5665590** | **-1736239** | **-529930** | **884256** | **5358711** | **-** | **-** | **-** | **-** | **-** | **5752104** | **4213702** | **-589805** | **-2453722** | **-1488814** | **-708433** | **-1324097** | **-3298218** |
|  | **M** | **64263247** | **8503560** | **26838173** | **37392140** | **26773131** | **50170309** | **-** | **-** | **-** | **-** | **-** | **103082850** | **1287314** | **-833110** | **-443690** | **351092** | **443023** | **276718** | **-66443** |
|  | **Mo** | **34043238** | **337811** | **4275256** | **4717302** | **6408134** | **30516956** | **-** | **-** | **-** | **-** | **-** | **13392966** | **139826** | **-346700** | **-498309** | **-153704** | **141615** | **-1898699** | **-194659** |
|  | **Q** | **42523924** | **-3114878** | **194917** | **8779601** | **9170229** | **35052132** | **-** | **-** | **-** | **-** | **-** | **26731150** | **-1553134** | **-1972422** | **-2502140** | **-1918232** | **-1933964** | **-1691551** | **-3015673** |
|  | **N** | **15029878** | **-3185894** | **-2149787** | **-463902** | **3472600** | **7441850** | **-** | **-** | **-** | **-** | **-** | **2490736** | **236555** | **-1245236** | **-1674993** | **-1127381** | **-1373134** | **-184878** | **-1342217** |
|  | **a** | **-650905** | **-2248134** | **-829844** | **-1901333** | **-1610484** | **-2617938** | **-998515** | **-988969** | **-57414** | **-749294** | **-705105** | **-** | **-** | **-** | **-** | **-** | **-** | **-** | **-** |
|  | **d** | **-41711** | **-1916664** | **-4234532** | **-2125175** | **-2196107** | **-4127906** | **-1342005** | **-3235772** | **-243037** | **-432118** | **-1978098** | **-** | **-** | **-** | **-** | **-** | **-** | **-** | **-** |
|  | **s** | **-1001037** | **-2154522** | **-1529937** | **-1909712** | **-1702102** | **-1896718** | **-1158618** | **-1396359** | **-164399** | **-2350263** | **-64103** | **-** | **-** | **-** | **-** | **-** | **-** | **-** | **-** |
|  | **b** | **-720347** | **-767706** | **-425441** | **-61364** | **-398156** | **-471060** | **-720626** | **-586666** | **-384898** | **-566663** | **-600993** | **-** | **-** | **-** | **-** | **-** | **-** | **-** | **-** |
|  | **sarcosine** | **9687027** | **7518567** | **33364423** | **21413926** | **26977116** | **11482507** | **14068128** | **11647479** | **32131303** | **-1549293** | **4888409** | **-** | **-** | **-** | **-** | **-** | **-** | **-** | **-** |

Cancer 3

|  |  | **P2** |  |  |  |  |  |  |  |  |  |  |  |  |  |  |  |  |  |  |
| --- | --- | --- | --- | --- | --- | --- | --- | --- | --- | --- | --- | --- | --- | --- | --- | --- | --- | --- | --- | --- |
|  |  | **G** | **E** | **K** | **Y** | **L** | **P** | **a** | **d** | **s** | **b** | **sarcosine** | **-** | **Ac** | **AcG** | **AcE** | **AcK** | **AcY** | **AcL** | **AcP** |
| **P1** | **G** | **400798** | **-23428** | **601885** | **699726** | **777927** | **-86763** | **1608** | **-294299** | **-273878** | **-170483** | **-215023** | **709335** | **-127685** | **-165382** | **-323643** | **-221314** | **-199286** | **-61297** | **-487965** |
|  | **E** | **-1016550** | **-603441** | **-436297** | **-160652** | **770917** | **-427946** | **-925445** | **-845873** | **-598917** | **-19124** | **-191632** | **912112** | **1189831** | **-1023202** | **-988345** | **-772051** | **-845272** | **-1120931** | **-901139** |
|  | **K** | **13053572** | **5266071** | **23105103** | **49389182** | **33096659** | **24254446** | **315963** | **-687953** | **1262903** | **-322873** | **1001290** | **44589635** | **-3328** | **-823671** | **-437990** | **4767176** | **-522474** | **276993** | **547102** |
|  | **Y** | **3813027** | **884180** | **4896140** | **5929330** | **4783187** | **4413306** | **69815** | **-51490** | **139600** | **-51376** | **67037** | **346053** | **42374** | **-101840** | **-117517** | **-164095** | **-20502** | **-107297** | **-57789** |
|  | **L** | **3883031** | **12202761** | **2454** | **21310021** | **27442651** | **3853965** | **262544** | **168249** | **558647** | **226972** | **262769** | **33709868** | **152200** | **29321** | **10717** | **321730** | **68733** | **86780** | **103541** |
|  | **P** | **8804901** | **12011328** | **30997193** | **8604169** | **13539637** | **24353944** | **1329078** | **3963507** | **7649071** | **152543** | **4768897** | **574917** | **-167697** | **943646** | **482869** | **6010115** | **2802314** | **3229045** | **5925717** |
|  | **F** | **2890533** | **1702072** | **3874534** | **5630489** | **1874013** | **5759887** | **-** | **-** | **-** | **-** | **-** | **6416772** | **644105** | **228285** | **188744** | **271391** | **157975** | **597984** | **355271** |
|  | **R** | **16552476** | **6463815** | **30872167** | **44741700** | **34499133** | **12432015** | **-** | **-** | **-** | **-** | **-** | **45290994** | **459975** | **1279341** | **745354** | **12434188** | **1316691** | **1764873** | **5300673** |
|  | **A** | **4331104** | **3428388** | **31721492** | **21897467** | **17972934** | **3706379** | **-** | **-** | **-** | **-** | **-** | **38767464** | **2194466** | **-60431** | **-29857** | **83462** | **154000** | **450873** | **55449** |
|  | **H** | **1629432** | **101158** | **606108** | **833670** | **785668** | **1434962** | **-** | **-** | **-** | **-** | **-** | **614766** | **-22803** | **-313549** | **-74471** | **-34846** | **20042** | **36079** | **-79240** |
|  | **W** | **2012764** | **202820** | **2853382** | **1857248** | **1402973** | **1198074** | **-** | **-** | **-** | **-** | **-** | **2640359** | **2838** | **12332** | **7965** | **-98222** | **33210** | **30810** | **728** |
|  | **S** | **505842** | **-967620** | **225254** | **77379** | **373508** | **-256988** | **-** | **-** | **-** | **-** | **-** | **1564312** | **2132489** | **20776** | **-169434** | **-75331** | **29016** | **149738** | **-117500** |
|  | **M** | **12605539** | **3188396** | **23484420** | **15454808** | **13586907** | **12148241** | **-** | **-** | **-** | **-** | **-** | **22670434** | **568107** | **29886** | **-65870** | **78596** | **176844** | **175242** | **76281** |
|  | **Mo** | **1859527** | **128943** | **2883834** | **1683311** | **1538014** | **1592743** | **-** | **-** | **-** | **-** | **-** | **3646238** | **61633** | **-46463** | **-84431** | **9970** | **99494** | **-298021** | **17489** |
|  | **Q** | **2385422** | **-527328** | **849014** | **1117904** | **1919998** | **669845** | **-** | **-** | **-** | **-** | **-** | **4587115** | **-248681** | **-389564** | **-525779** | **-519808** | **-574819** | **-346774** | **-557290** |
|  | **N** | **432984** | **-846933** | **88721** | **247566** | **2083132** | **205400** | **-** | **-** | **-** | **-** | **-** | **1110319** | **359831** | **-171095** | **-322200** | **-175540** | **-293006** | **13541** | **-131791** |
|  | **a** | **125835** | **-315436** | **-134203** | **-310999** | **-184367** | **-514319** | **-185930** | **-102802** | **82857** | **-113894** | **-94258** | **-** | **-** | **-** | **-** | **-** | **-** | **-** | **-** |
|  | **d** | **259622** | **-677911** | **-806132** | **-387691** | **325344** | **-515301** | **-161170** | **-1148800** | **-36697** | **-124772** | **-488448** | **-** | **-** | **-** | **-** | **-** | **-** | **-** | **-** |
|  | **s** | **-277057** | **-384443** | **-268706** | **-375873** | **-247031** | **-496896** | **-198506** | **-296513** | **-2847** | **-455301** | **-523442** | **-** | **-** | **-** | **-** | **-** | **-** | **-** | **-** |
|  | **b** | **-170316** | **-191215** | **-75464** | **-13588** | **-66774** | **-96754** | **-153837** | **-114419** | **-64305** | **-97795** | **-123736** | **-** | **-** | **-** | **-** | **-** | **-** | **-** | **-** |
|  | **sarcosine** | **5132233** | **4623878** | **18369337** | **7968094** | **10233310** | **5143561** | **7394839** | **8591532** | **19815830** | **-342055** | **2701102** | **-** | **-** | **-** | **-** | **-** | **-** | **-** | **-** |

Normal 3

|  |  | **P2** |  |  |  |  |  |  |  |  |  |  |  |  |  |  |  |  |  |  |
| --- | --- | --- | --- | --- | --- | --- | --- | --- | --- | --- | --- | --- | --- | --- | --- | --- | --- | --- | --- | --- |
|  |  | **G** | **E** | **K** | **Y** | **L** | **P** | **a** | **d** | **s** | **b** | **sarcosine** | **-** | **Ac** | **AcG** | **AcE** | **AcK** | **AcY** | **AcL** | **AcP** |
| **P1** | **G** | **1120310** | **115669** | **260189** | **1063104** | **1514679** | **1766446** | **99533** | **2777** | **516665** | **-3010** | **231798** | **2098941** | **-36007** | **380325** | **449288** | **377234** | **260145** | **564529** | **-195407** |
|  | **E** | **275412** | **308078** | **-441830** | **824216** | **699574** | **634805** | **197254** | **230431** | **355930** | **5651** | **132635** | **1475943** | **1083886** | **-662222** | **-195366** | **-277457** | **-63380** | **-537833** | **72426** |
|  | **K** | **20480340** | **3800660** | **6725900** | **19211899** | **11400738** | **37353676** | **3435981** | **-1174713** | **2999799** | **-89963** | **5820122** | **24206139** | **557353** | **4990032** | **4882400** | **38453935** | **3842367** | **4621146** | **14321629** |
|  | **Y** | **8099548** | **615570** | **2056283** | **4122289** | **3296464** | **7512422** | **327527** | **134168** | **495954** | **72716** | **367158** | **338463** | **348047** | **38107** | **107732** | **23317** | **105971** | **169400** | **152620** |
|  | **L** | **5511655** | **4807540** | **17467** | **12088055** | **11132283** | **9432593** | **1081545** | **552844** | **1293562** | **1122495** | **964086** | **23216978** | **314526** | **174695** | **168616** | **280380** | **156303** | **180188** | **201436** |
|  | **P** | **20281232** | **14008427** | **34236889** | **11149872** | **15865222** | **24539891** | **2844681** | **8421881** | **14058207** | **206992** | **4775665** | **361453** | **-84346** | **631804** | **178267** | **3621246** | **2059850** | **2281427** | **2622193** |
|  | **F** | **5975500** | **2313381** | **2313628** | **5593910** | **1751203** | **6413931** | **-** | **-** | **-** | **-** | **-** | **7646707** | **784385** | **833443** | **848090** | **1042211** | **560805** | **2050136** | **1508542** |
|  | **R** | **52485181** | **4347553** | **8243990** | **18271859** | **15562292** | **38192842** | **-** | **-** | **-** | **-** | **-** | **39427206** | **1639906** | **10938777** | **5030898** | **65901624** | **6595459** | **12147392** | **36155512** |
|  | **A** | **8181674** | **3755824** | **12582136** | **10002606** | **10109045** | **6333842** | **-** | **-** | **-** | **-** | **-** | **30135398** | **4009395** | **452213** | **283669** | **501920** | **-249055** | **492990** | **-225442** |
|  | **H** | **5409322** | **240305** | **563570** | **1064186** | **1436573** | **5260583** | **-** | **-** | **-** | **-** | **-** | **721033** | **181829** | **-39534** | **47355** | **61481** | **50214** | **172007** | **-84363** |
|  | **W** | **6422288** | **209211** | **1178882** | **1412781** | **995992** | **3393944** | **-** | **-** | **-** | **-** | **-** | **2374608** | **46125** | **29089** | **36463** | **110453** | **79187** | **94086** | **42205** |
|  | **S** | **2055756** | **-764330** | **-151372** | **361266** | **1242977** | **1483389** | **-** | **-** | **-** | **-** | **-** | **1650524** | **2509755** | **304263** | **574593** | **136993** | **671652** | **882999** | **1026432** |
|  | **M** | **15142133** | **1289078** | **13139156** | **9759160** | **9880600** | **12583307** | **-** | **-** | **-** | **-** | **-** | **47541474** | **686297** | **484698** | **193636** | **198077** | **114672** | **317933** | **130650** |
|  | **Mo** | **6431352** | **94508** | **1371454** | **903698** | **1739993** | **5565416** | **-** | **-** | **-** | **-** | **-** | **4918286** | **96501** | **14361** | **-45829** | **-19826** | **94526** | **439217** | **143840** |
|  | **Q** | **10870568** | **1519450** | **2094989** | **2499012** | **3033636** | **7399380** | **-** | **-** | **-** | **-** | **-** | **8900426** | **-368517** | **14123** | **-140185** | **-18466** | **225796** | **177262** | **79816** |
|  | **N** | **4166238** | **1273900** | **1420007** | **1494503** | **4824687** | **1918444** | **-** | **-** | **-** | **-** | **-** | **1242025** | **815381** | **-128202** | **-176604** | **119991** | **8062** | **274194** | **311369** |
|  | **a** | **760539** | **346421** | **418484** | **149860** | **892071** | **-28189** | **71342** | **136378** | **306706** | **35707** | **205256** | **-** | **-** | **-** | **-** | **-** | **-** | **-** | **-** |
|  | **d** | **1073512** | **879904** | **-505557** | **49073** | **1965151** | **199674** | **1178165** | **-294963** | **7949** | **-23236** | **-70263** | **-** | **-** | **-** | **-** | **-** | **-** | **-** | **-** |
|  | **s** | **68807** | **69225** | **83065** | **27959** | **101458** | **67922** | **62518** | **-165070** | **41304** | **-97064** | **15181** | **-** | **-** | **-** | **-** | **-** | **-** | **-** | **-** |
|  | **b** | **-5230** | **26209** | **-91720** | **29985** | **38065** | **22950** | **53695** | **46215** | **79471** | **132410** | **19933** | **-** | **-** | **-** | **-** | **-** | **-** | **-** | **-** |
|  | **sarcosine** | **11225399** | **7320838** | **31439262** | **14676994** | **16651173** | **8569027** | **14106186** | **19582981** | **35926828** | **-171982** | **5186862** | **-** | **-** | **-** | **-** | **-** | **-** | **-** | **-** |

Cancer 4

|  |  | **P2** |  |  |  |  |  |  |  |  |  |  |  |  |  |  |  |  |  |  |
| --- | --- | --- | --- | --- | --- | --- | --- | --- | --- | --- | --- | --- | --- | --- | --- | --- | --- | --- | --- | --- |
|  |  | **G** | **E** | **K** | **Y** | **L** | **P** | **a** | **d** | **s** | **b** | **sarcosine** | **-** | **Ac** | **AcG** | **AcE** | **AcK** | **AcY** | **AcL** | **AcP** |
| **P1** | **G** | **2087988** | **80530** | **673904** | **2051346** | **2517300** | **3128518** | **170977** | **-163770** | **295141** | **-104935** | **3677** | **1686932** | **-148013** | **-174971** | **-323545** | **-274582** | **-34390** | **-55830** | **-805453** |
|  | **E** | **663421** | **-266495** | **30225** | **433820** | **1519315** | **409183** | **-566686** | **-703300** | **-290366** | **-6177** | **-223396** | **2173551** | **2675911** | **-1394366** | **-1014701** | **-965928** | **-938936** | **-1252856** | **-1120281** |
|  | **K** | **43308610** | **6239706** | **19475770** | **43109679** | **36636076** | **61446147** | **6252880** | **-757024** | **6611598** | **51986** | **13008005** | **46797394** | **165199** | **1332588** | **769414** | **19167542** | **1606010** | **2363447** | **7147314** |
|  | **Y** | **18514745** | **2108723** | **7200797** | **11871899** | **7999525** | **15937122** | **831984** | **-62937** | **1300368** | **32547** | **629143** | **16787336** | **187938** | **-14316** | **-165726** | **-178262** | **36083** | **-77992** | **-41591** |
|  | **L** | **12699023** | **2463892** | **20098568** | **29235653** | **34048095** | **18188238** | **777124** | **81030** | **1400837** | **764853** | **503528** | **56975437** | **288838** | **49824** | **-13133** | **430480** | **182561** | **237453** | **208138** |
|  | **P** | **22761833** | **19675147** | **47450697** | **15979528** | **24013836** | **35333692** | **1816795** | **4893500** | **11532291** | **253208** | **7801192** | **939713** | **-78115** | **1545400** | **1011330** | **10944979** | **5941014** | **6695504** | **9212447** |
|  | **F** | **12479306** | **2866703** | **4965580** | **8730905** | **5049779** | **11641072** | **-** | **-** | **-** | **-** | **-** | **13652178** | **1844694** | **182268** | **167656** | **182943** | **206267** | **697376** | **283189** |
|  | **R** | **103250362** | **9163750** | **32530619** | **54363352** | **41672263** | **56465581** | **-** | **-** | **-** | **-** | **-** | **58597896** | **1117337** | **4933371** | **2752429** | **41750512** | **4616828** | **6328601** | **22984673** |
|  | **A** | **18333744** | **6288089** | **33896151** | **34080426** | **25100689** | **13578264** | **-** | **-** | **-** | **-** | **-** | **50503125** | **3292574** | **-12424** | **-178892** | **159422** | **351535** | **720247** | **51111** |
|  | **H** | **17627385** | **199853** | **870482** | **1693557** | **2148359** | **12564419** | **-** | **-** | **-** | **-** | **-** | **1357744** | **-7232** | **-429434** | **-121252** | **-68739** | **54336** | **127646** | **-187916** |
|  | **W** | **14218835** | **874360** | **4030303** | **4327883** | **2652366** | **7149932** | **-** | **-** | **-** | **-** | **-** | **6984833** | **54303** | **56695** | **3534** | **-126051** | **95380** | **89531** | **24452** |
|  | **S** | **5831804** | **-1618842** | **460769** | **1080306** | **1871610** | **4356122** | **-** | **-** | **-** | **-** | **-** | **2839734** | **4440640** | **721334** | **-573413** | **-201117** | **104106** | **-75351** | **-953709** |
|  | **M** | **32414882** | **6628916** | **36253342** | **33572651** | **55374534** | **760963** | **-** | **-** | **-** | **-** | **-** | **83545449** | **1193388** | **28133** | **-28455** | **176365** | **544711** | **497719** | **336775** |
|  | **Mo** | **17503004** | **373273** | **3840530** | **139603** | **3775642** | **17412247** | **-** | **-** | **-** | **-** | **-** | **8684747** | **541570** | **-132159** | **-140688** | **24010** | **277790** | **-549249** | **91903** |
|  | **Q** | **23884957** | **-1020785** | **2208893** | **6317432** | **7092582** | **22376335** | **-** | **-** | **-** | **-** | **-** | **13807934** | **-242262** | **-457323** | **-732158** | **-637726** | **-448384** | **-197426** | **-698517** |
|  | **N** | **9393065** | **-1093031** | **-43215** | **1036044** | **4795664** | **3999511** | **-** | **-** | **-** | **-** | **-** | **1620311** | **740821** | **-147372** | **-529025** | **-205350** | **-381168** | **46926** | **-405134** |
|  | **a** | **107978** | **-472839** | **-47255** | **-330127** | **174470** | **-643386** | **-249469** | **-241947** | **139268** | **-174440** | **-126866** | **-** | **-** | **-** | **-** | **-** | **-** | **-** | **-** |
|  | **d** | **412031** | **-628826** | **-1078849** | **-610858** | **257710** | **-1359264** | **31758** | **-1130001** | **-42916** | **-139987** | **-450940** | **-** | **-** | **-** | **-** | **-** | **-** | **-** | **-** |
|  | **s** | **-217206** | **-557017** | **-376102** | **-416687** | **-308955** | **-476787** | **-330971** | **-436275** | **-16462** | **-567487** | **-792759** | **-** | **-** | **-** | **-** | **-** | **-** | **-** | **-** |
|  | **b** | **-219209** | **-252180** | **-26075** | **6478** | **-45767** | **-166457** | **-163790** | **-185083** | **-109766** | **-166238** | **-130085** | **-** | **-** | **-** | **-** | **-** | **-** | **-** | **-** |
|  | **sarcosine** | **6931972** | **6411428** | **29082716** | **14900670** | **16752135** | **7738626** | **10357012** | **10523532** | **25714916** | **-379067** | **2990450** | **-** | **-** | **-** | **-** | **-** | **-** | **-** | **-** |

Normal 4

|  |  | **P2** |  |  |  |  |  |  |  |  |  |  |  |  |  |  |  |  |  |  |
| --- | --- | --- | --- | --- | --- | --- | --- | --- | --- | --- | --- | --- | --- | --- | --- | --- | --- | --- | --- | --- |
|  |  | **G** | **E** | **K** | **Y** | **L** | **P** | **a** | **d** | **s** | **b** | **sarcosine** | **-** | **Ac** | **AcG** | **AcE** | **AcK** | **AcY** | **AcL** | **AcP** |
| **P1** | **G** | **3554578** | **73089** | **2689701** | **5210192** | **4040479** | **3832528** | **188915** | **-253832** | **329970** | **-133144** | **73099** | **5732396** | **-241182** | **-278158** | **-295132** | **-231618** | **-86860** | **-55741** | **-1212401** |
|  | **E** | **1818832** | **-251640** | **380067** | **170960** | **1473795** | **620313** | **-916853** | **-931209** | **-731902** | **-66130** | **-492162** | **904733** | **1728987** | **-1945322** | **-1633561** | **-1458692** | **-1267588** | **-1959673** | **-1892230** |
|  | **K** | **48490814** | **3655001** | **31486982** | **41832736** | **33445906** | **64295925** | **7459106** | **-999785** | **7064519** | **-176301** | **13581079** | **53338928** | **20794** | **-126882** | **340038** | **9768324** | **124837** | **802427** | **977355** |
|  | **Y** | **30047111** | **2248523** | **16943536** | **27064910** | **15129562** | **23244942** | **1102014** | **41584** | **1595142** | **98848** | **856501** | **34238065** | **-382611** | **-73583** | **-190561** | **-232393** | **97082** | **5794** | **-56780** |
|  | **L** | **16446954** | **947518** | **20102637** | **35301660** | **34297241** | **21090191** | **1235888** | **266441** | **1850378** | **1310100** | **764190** | **71259066** | **224949** | **16421** | **484** | **408149** | **150917** | **262745** | **311355** |
|  | **P** | **8840928** | **6790607** | **26061565** | **6817933** | **9836831** | **13447807** | **2559935** | **6893759** | **13463801** | **42149** | **2577073** | **890298** | **-229156** | **1093565** | **772386** | **9193188** | **5273080** | **6849831** | **8552818** |
|  | **F** | **20127358** | **2810371** | **7641924** | **15492801** | **7378681** | **16971502** | **-** | **-** | **-** | **-** | **-** | **23318094** | **1568617** | **422854** | **309896** | **227242** | **419047** | **2026033** | **1050601** |
|  | **R** | **110704774** | **3147607** | **35785432** | **62040239** | **37369108** | **54053351** | **-** | **-** | **-** | **-** | **-** | **66660053** | **996764** | **2548246** | **1399757** | **23585718** | **2103609** | **3917116** | **12467893** |
|  | **A** | **39626062** | **3426982** | **49237599** | **59123143** | **37497242** | **19748079** | **-** | **-** | **-** | **-** | **-** | **86269390** | **2651895** | **205691** | **-90500** | **247669** | **181056** | **946190** | **-568318** |
|  | **H** | **17789031** | **167658** | **1281852** | **2037589** | **2241646** | **14699120** | **-** | **-** | **-** | **-** | **-** | **2270766** | **-34055** | **-718744** | **-101854** | **-75246** | **43628** | **152547** | **-304582** |
|  | **W** | **22072172** | **706216** | **8912903** | **9681796** | **5063248** | **11163364** | **-** | **-** | **-** | **-** | **-** | **14221453** | **29782** | **31117** | **-10552** | **-192460** | **96193** | **94252** | **40834** |
|  | **S** | **7458733** | **-2264345** | **1476932** | **2911076** | **2825493** | **5234958** | **-** | **-** | **-** | **-** | **-** | **6814851** | **3166365** | **890328** | **-997533** | **-232021** | **458195** | **91362** | **-719964** |
|  | **M** | **49038743** | **4177764** | **50202463** | **59136368** | **77101871** | **829052** | **-** | **-** | **-** | **-** | **-** | **126343662** | **975062** | **-154693** | **77370** | **108478** | **311288** | **354635** | **226170** |
|  | **Mo** | **21314951** | **180664** | **6971550** | **248765** | **8976946** | **25814525** | **-** | **-** | **-** | **-** | **-** | **23560056** | **804548** | **-221505** | **-271523** | **-40179** | **126431** | **-851944** | **-9976** |
|  | **Q** | **29044634** | **-1349885** | **10391036** | **21509336** | **19669014** | **23912747** | **-** | **-** | **-** | **-** | **-** | **42724239** | **-858118** | **-1087842** | **-1214967** | **-1029251** | **-1099444** | **-468805** | **-1302504** |
|  | **N** | **10345969** | **-2152459** | **-455970** | **1252416** | **4515920** | **4973086** | **-** | **-** | **-** | **-** | **-** | **2394117** | **499545** | **-525098** | **-914586** | **-584177** | **-675244** | **-40869** | **-762787** |
|  | **a** | **273426** | **-733896** | **-51910** | **-494654** | **106127** | **-978980** | **-283020** | **-344324** | **160763** | **-214500** | **-231042** | **-** | **-** | **-** | **-** | **-** | **-** | **-** | **-** |
|  | **d** | **559597** | **-904145** | **-1731382** | **-968832** | **174367** | **-1509667** | **-164975** | **-1658428** | **-88359** | **-220424** | **-802910** | **-** | **-** | **-** | **-** | **-** | **-** | **-** | **-** |
|  | **s** | **-322346** | **-641785** | **-487874** | **-332805** | **-147956** | **-566146** | **-539889** | **-770168** | **-36133** | **-993180** | **-1104043** | **-** | **-** | **-** | **-** | **-** | **-** | **-** | **-** |
|  | **b** | **-284731** | **-352667** | **-57099** | **75221** | **-12512** | **-173583** | **-193815** | **-261990** | **-75822** | **-152438** | **-152062** | **-** | **-** | **-** | **-** | **-** | **-** | **-** | **-** |
|  | **sarcosine** | **9203211** | **6471194** | **29160437** | **15556922** | **18550526** | **5947314** | **13631434** | **14749029** | **34212552** | **-617293** | **4349851** | **-** | **-** | **-** | **-** | **-** | **-** | **-** | **-** |

Cancer 5

|  |  | **P2** |  |  |  |  |  |  |  |  |  |  |  |  |  |  |  |  |  |  |
| --- | --- | --- | --- | --- | --- | --- | --- | --- | --- | --- | --- | --- | --- | --- | --- | --- | --- | --- | --- | --- |
|  |  | **G** | **E** | **K** | **Y** | **L** | **P** | **a** | **d** | **s** | **b** | **sarcosine** | **-** | **Ac** | **AcG** | **AcE** | **AcK** | **AcY** | **AcL** | **AcP** |
| **P1** | **G** | **833994** | **36624** | **570038** | **1474035** | **1117603** | **529936** | **166776** | **-251339** | **-71851** | **-130958** | **-131574** | **1263972** | **-147575** | **-89346** | **-271836** | **-341557** | **-197254** | **-176288** | **-625611** |
|  | **E** | **-881655** | **-337760** | **-184053** | **254462** | **1842442** | **-650662** | **-898440** | **-864034** | **-776292** | **-36268** | **-392415** | **1795916** | **3024470** | **-1076549** | **-1078131** | **-904215** | **-988250** | **-1361148** | **-1130247** |
|  | **K** | **21120307** | **4912645** | **22848416** | **45933755** | **37320804** | **32117319** | **1361663** | **-1144028** | **2497810** | **-388416** | **3630219** | **48829291** | **325176** | **2778092** | **2052439** | **28836797** | **2314968** | **3582163** | **12176381** |
|  | **Y** | **9431986** | **1444348** | **5639287** | **12057305** | **6926867** | **7476296** | **294203** | **-35638** | **416633** | **-25370** | **219394** | **16189566** | **221484** | **-30408** | **-176793** | **-153189** | **10334** | **-191351** | **-130819** |
|  | **L** | **7483115** | **2133977** | **27420498** | **38447336** | **47026570** | **11585255** | **598689** | **14424** | **871790** | **498588** | **360978** | **72994532** | **401100** | **44244** | **95441** | **611010** | **203487** | **245811** | **169320** |
|  | **P** | **25072098** | **21298601** | **46411625** | **18233425** | **25702746** | **34885589** | **1758585** | **4731824** | **10515677** | **271496** | **7875011** | **1501852** | **-127383** | **1948260** | **1337208** | **14583617** | **10374289** | **10738913** | **14836530** |
|  | **F** | **9023120** | **2625255** | **5328351** | **13460701** | **7425089** | **10781393** | **-** | **-** | **-** | **-** | **-** | **18136216** | **2304152** | **314258** | **2615** | **57436** | **199206** | **320543** | **484058** |
|  | **R** | **48374536** | **8080518** | **24711861** | **55273094** | **40982630** | **23994762** | **-** | **-** | **-** | **-** | **-** | **57544660** | **1419941** | **8688151** | **4792526** | **57064071** | **6978486** | **9601524** | **38372294** |
|  | **A** | **8074287** | **4100994** | **29253423** | **29132423** | **20872727** | **5550261** | **-** | **-** | **-** | **-** | **-** | **44381340** | **4399945** | **-201163** | **-233398** | **285075** | **439558** | **692159** | **202982** |
|  | **H** | **5383111** | **121036** | **852542** | **1605143** | **1619807** | **3555208** | **-** | **-** | **-** | **-** | **-** | **1574530** | **37439** | **-586843** | **-100737** | **-66471** | **-1885** | **16640** | **-165131** |
|  | **W** | **7116328** | **800568** | **5130375** | **6308953** | **4109612** | **3366495** | **-** | **-** | **-** | **-** | **-** | **10804064** | **77527** | **58884** | **11936** | **-111414** | **126125** | **108059** | **5454** |
|  | **S** | **1119437** | **-1861728** | **-315596** | **352302** | **952418** | **81164** | **-** | **-** | **-** | **-** | **-** | **2147050** | **4909501** | **35764** | **-526078** | **-146360** | **-122171** | **-312914** | **-664794** |
|  | **M** | **19391287** | **4602250** | **31349801** | **35337772** | **41376940** | **120235** | **-** | **-** | **-** | **-** | **-** | **82103147** | **1593369** | **118170** | **-170782** | **180001** | **423427** | **319192** | **99847** |
|  | **Mo** | **4668374** | **207473** | **2936093** | **32700** | **2435494** | **5871934** | **-** | **-** | **-** | **-** | **-** | **5453581** | **210968** | **-123296** | **-180026** | **-1347** | **155362** | **-537178** | **12215** |
|  | **Q** | **6197329** | **-744087** | **889740** | **3457557** | **3551858** | **4607529** | **-** | **-** | **-** | **-** | **-** | **8761608** | **-104572** | **-665411** | **-875547** | **-646322** | **-604321** | **-444595** | **-752190** |
|  | **N** | **1814206** | **-1317307** | **-645118** | **754267** | **4267792** | **523654** | **-** | **-** | **-** | **-** | **-** | **1467059** | **798821** | **-161155** | **-658226** | **-346407** | **-380312** | **-81484** | **-497468** |
|  | **a** | **220143** | **-430308** | **-101839** | **-331775** | **108698** | **-539514** | **-246409** | **-272469** | **40575** | **-170377** | **-115819** | **-** | **-** | **-** | **-** | **-** | **-** | **-** | **-** |
|  | **d** | **313828** | **-864601** | **-1676691** | **-590043** | **368560** | **-1091679** | **-90186** | **-1114295** | **-50917** | **-146557** | **-430247** | **-** | **-** | **-** | **-** | **-** | **-** | **-** | **-** |
|  | **s** | **-231108** | **-625687** | **-463533** | **-463693** | **-243561** | **-551906** | **-446880** | **-648666** | **-33378** | **-724460** | **-691446** | **-** | **-** | **-** | **-** | **-** | **-** | **-** | **-** |
|  | **b** | **-213507** | **-203711** | **-55830** | **67469** | **50143** | **-89956** | **-192759** | **-167827** | **-29475** | **-152434** | **-128790** | **-** | **-** | **-** | **-** | **-** | **-** | **-** | **-** |
|  | **sarcosine** | **6736904** | **5632796** | **27245827** | **15159134** | **17693635** | **7564552** | **8432938** | **10377276** | **23157255** | **-404524** | **2986658** | **-** | **-** | **-** | **-** | **-** | **-** | **-** | **-** |

Normal 5

|  |  | **P2** |  |  |  |  |  |  |  |  |  |  |  |  |  |  |  |  |  |  |
| --- | --- | --- | --- | --- | --- | --- | --- | --- | --- | --- | --- | --- | --- | --- | --- | --- | --- | --- | --- | --- |
|  |  | **G** | **E** | **K** | **Y** | **L** | **P** | **a** | **d** | **s** | **b** | **sarcosine** | **-** | **Ac** | **AcG** | **AcE** | **AcK** | **AcY** | **AcL** | **AcP** |
| **P1** | **G** | **2204279** | **39015** | **1000863** | **3439347** | **1775871** | **2202745** | **114405** | **-97751** | **142362** | **-29057** | **76566** | **3620936** | **-128023** | **-215604** | **-215678** | **-170117** | **-188504** | **-17560** | **-745272** |
|  | **E** | **558886** | **-432683** | **-219011** | **242788** | **705844** | **313178** | **-560108** | **-548753** | **-216577** | **-37976** | **-253043** | **703383** | **1246044** | **-835536** | **-543488** | **-735575** | **-647222** | **-1126427** | **-476492** |
|  | **K** | **27725379** | **1514823** | **14874422** | **32891366** | **23411862** | **36224365** | **4384706** | **-1120517** | **4161756** | **259037** | **7651393** | **34031097** | **47775** | **347032** | **-337696** | **7255112** | **256887** | **751357** | **2247203** |
|  | **Y** | **19350202** | **1452979** | **12002982** | **18949853** | **9636559** | **14270248** | **656610** | **32554** | **899720** | **69824** | **535852** | **22587762** | **17894** | **152159** | **56326** | **-42269** | **204908** | **-21629** | **60867** |
|  | **L** | **10403060** | **631983** | **14116725** | **24945243** | **24003767** | **13447089** | **804076** | **119614** | **905914** | **763381** | **534088** | **46308675** | **138990** | **9399** | **-1132** | **282190** | **168383** | **189448** | **274175** |
|  | **P** | **4757494** | **3839137** | **18227169** | **4528353** | **6564539** | **8472058** | **1408679** | **3823703** | **7727708** | **45598** | **1528508** | **535838** | **1722** | **672539** | **439261** | **5258481** | **3390095** | **4481879** | **5826975** |
|  | **F** | **14184872** | **1677180** | **3899793** | **9848269** | **5645626** | **10766491** | **-** | **-** | **-** | **-** | **-** | **15039162** | **1057136** | **296553** | **252829** | **55870** | **203783** | **1119373** | **247257** |
|  | **R** | **65834866** | **2163396** | **23475819** | **43096230** | **25638661** | **33673432** | **-** | **-** | **-** | **-** | **-** | **42472197** | **622075** | **2727310** | **1214597** | **16925732** | **1986544** | **3048828** | **11258398** |
|  | **A** | **23513823** | **2371745** | **31531894** | **36784319** | **23842780** | **10972810** | **-** | **-** | **-** | **-** | **-** | **49591055** | **2014550** | **134312** | **-12548** | **90289** | **281703** | **614076** | **-57666** |
|  | **H** | **9767195** | **180021** | **860959** | **1448153** | **1587735** | **8294194** | **-** | **-** | **-** | **-** | **-** | **1455365** | **7350** | **-335477** | **-54349** | **-51016** | **7919** | **80208** | **-93739** |
|  | **W** | **12678917** | **419541** | **5765580** | **6045345** | **3136661** | **6671911** | **-** | **-** | **-** | **-** | **-** | **8890231** | **31242** | **31752** | **14277** | **-53199** | **70273** | **90705** | **30076** |
|  | **S** | **4402980** | **-1157805** | **1245368** | **1977969** | **1759939** | **3090445** | **-** | **-** | **-** | **-** | **-** | **4098474** | **2024975** | **387939** | **76703** | **428161** | **367481** | **200165** | **-219677** |
|  | **M** | **30740837** | **2653055** | **31710026** | **35764894** | **43910877** | **392740** | **-** | **-** | **-** | **-** | **-** | **77621821** | **701608** | **-119581** | **-48839** | **75778** | **330618** | **229456** | **158947** |
|  | **Mo** | **12371075** | **120888** | **4646527** | **123481** | **5931332** | **15287685** | **-** | **-** | **-** | **-** | **-** | **13455010** | **428365** | **-35747** | **-100864** | **-29283** | **67833** | **20732** | **56507** |
|  | **Q** | **16475868** | **133052** | **6755882** | **13684209** | **12935244** | **13898377** | **-** | **-** | **-** | **-** | **-** | **26115005** | **-424125** | **-319368** | **-231273** | **-403814** | **-298725** | **-206374** | **-507010** |
|  | **N** | **6754638** | **-604959** | **180345** | **724854** | **2170883** | **2905161** | **-** | **-** | **-** | **-** | **-** | **1406216** | **333691** | **-57799** | **-446836** | **-222126** | **-234467** | **96216** | **-229961** |
|  | **a** | **-45966** | **-98974** | **67963** | **-362167** | **-175032** | **-440776** | **-126635** | **-150112** | **28928** | **-160079** | **-13833** | **-** | **-** | **-** | **-** | **-** | **-** | **-** | **-** |
|  | **d** | **320971** | **-394089** | **-268160** | **-342792** | **691905** | **140750** | **69362** | **-764463** | **-47473** | **-97522** | **-343040** | **-** | **-** | **-** | **-** | **-** | **-** | **-** | **-** |
|  | **s** | **-85145** | **-397075** | **-502824** | **-417693** | **-202401** | **-409375** | **-372296** | **-256150** | **-19271** | **-612972** | **-162319** | **-** | **-** | **-** | **-** | **-** | **-** | **-** | **-** |
|  | **b** | **-61789** | **-48099** | **-11477** | **70853** | **16769** | **-48885** | **-112301** | **-193405** | **-84397** | **-24456** | **-42098** | **-** | **-** | **-** | **-** | **-** | **-** | **-** | **-** |
|  | **sarcosine** | **5522816** | **3549430** | **16207755** | **9672372** | **10996870** | **3271255** | **7801401** | **8692865** | **19489823** | **-332106** | **2334705** | **-** | **-** | **-** | **-** | **-** | **-** | **-** | **-** |

## Supplementary Table 2. The result of analysis of primary probe selection

## Supplementary Table 2A. The median ratio of FI increase between cancer and non-cancer lysate. Orange: the ratio ≥ 90th percentile.


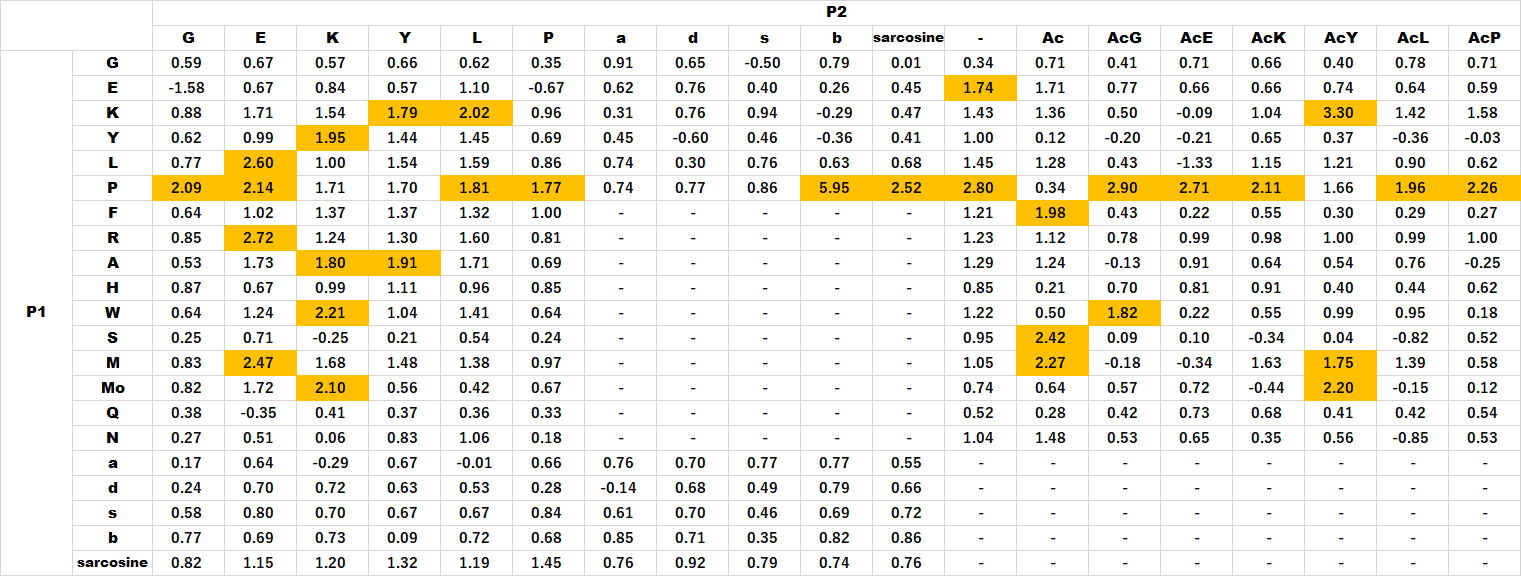


##
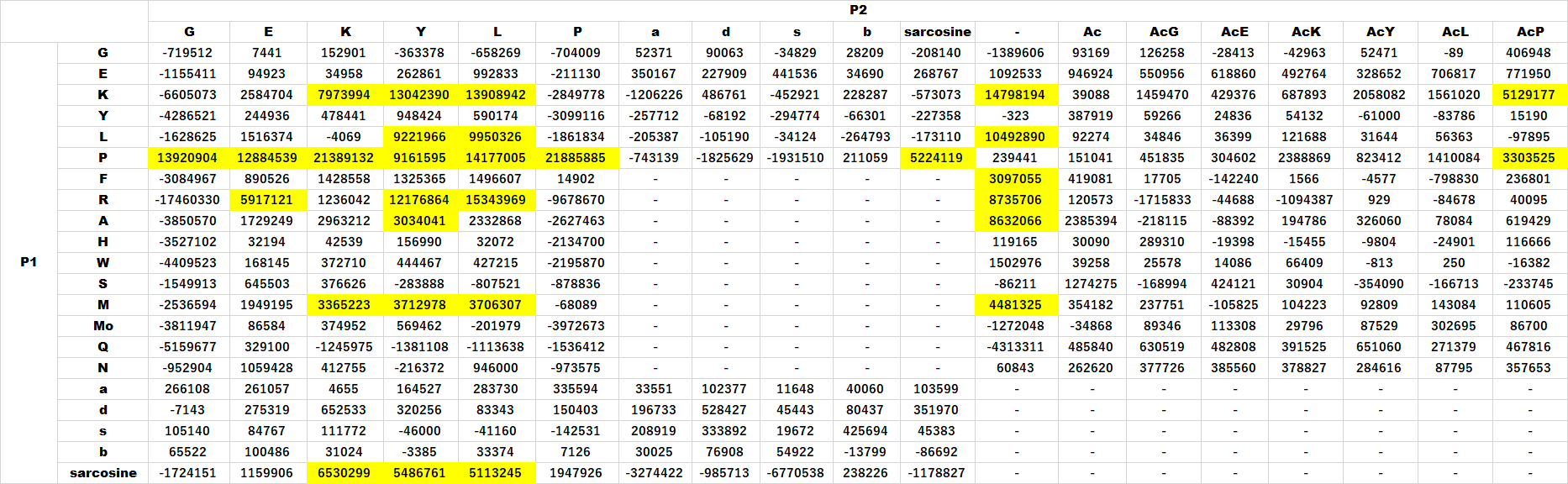
Supplementary Table 2B. The median difference of FI increase between cancer and non-cancer lysate. Yellow: the difference ≥ 90th percentile.

## Supplementary table 3. The patient’s demographic characteristics of primary and secondary probe selection

## Supplementary table 3A. The patient’s demographic characteristics of primary probe selection

| Patient | Age (y) | Sex | Preoperative CA19-9 (IU/mL) | Surgical procedures | Histological type |
| --- | --- | --- | --- | --- | --- |
| 1 | 73 | F | 78  13310  300 | DP | por |
| 2 | 81 | M | 13310 | PD | tub2-por |
| 3 | 67 | M | 300 | PD | tub2 > por |
| 4 | 81 | M | 141 | DP | tub1 > tub2 > por |
| 5 | 75 | M | 21 | DP | tub1 > por |

## PD: pancreaticoduodenectomy, DP: distal pancreatectomy, tub1/tub2: well/moderately differentiated tubular adenocarcinoma, por: poorly differentiated adenocarcinoma

## Supplementary table 3B. The patient’s demographic characteristics of secondary probe selection

| Patient | Age (y) | Sex | Preoperative  CA19-9 (IU/mL) | Surgical procedures | Collected sample | Histological type |
| --- | --- | --- | --- | --- | --- | --- |
| 1 | 83 | F | 364 | DP | Both | tub1 |
| 2 | 75 | M | 161 | DP | Both | tub1, tub2 |
| 3 | 66 | F | 54 | PD | Both | tub1, tub2 > por |
| 4 | 80 | F | 408 | PD | Both | tub1 > tub2 > por |
| 5 | 78 | M | 52 | DP | Tumor | tub1 > tub2 |
| 6 | 70 | M | 11 | PD | Both | IPMC |
| 7 | 81 | F | 34 | PD | Both | mod > por |
| 8 | 55 | M | 28 | PD | Normal | Autoimmune pancreatitis |
| 9 | 69 | M | 416 | DP | Both | mod > por |
| 10 | 86 | M | 132 | PD | Both | IPMC |
| 11 | 83 | M | 1440 | PD | Both | tub2 > tub1, por |

## PD: pancreaticoduodenectomy, DP: distal pancreatectomy, tub1/tub2: well/moderately differentiated tubular adenocarcinoma, por: poorly differentiated adenocarcinoma, IPMC: intraductal papillary mucinous carcinoma

# Supplementary Figure

## Supplementary Figure 1. Fluorescence imaging of the other resected specimen.


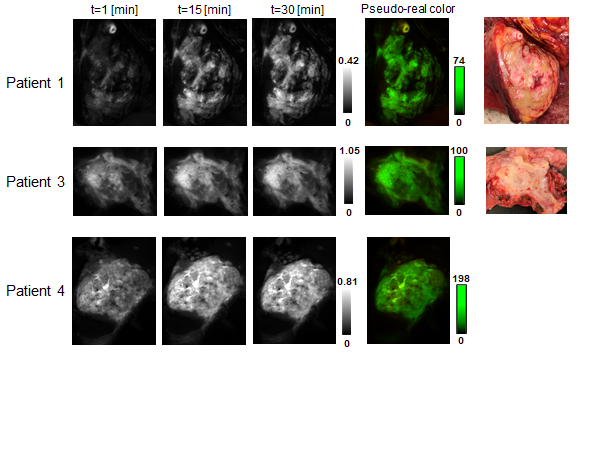


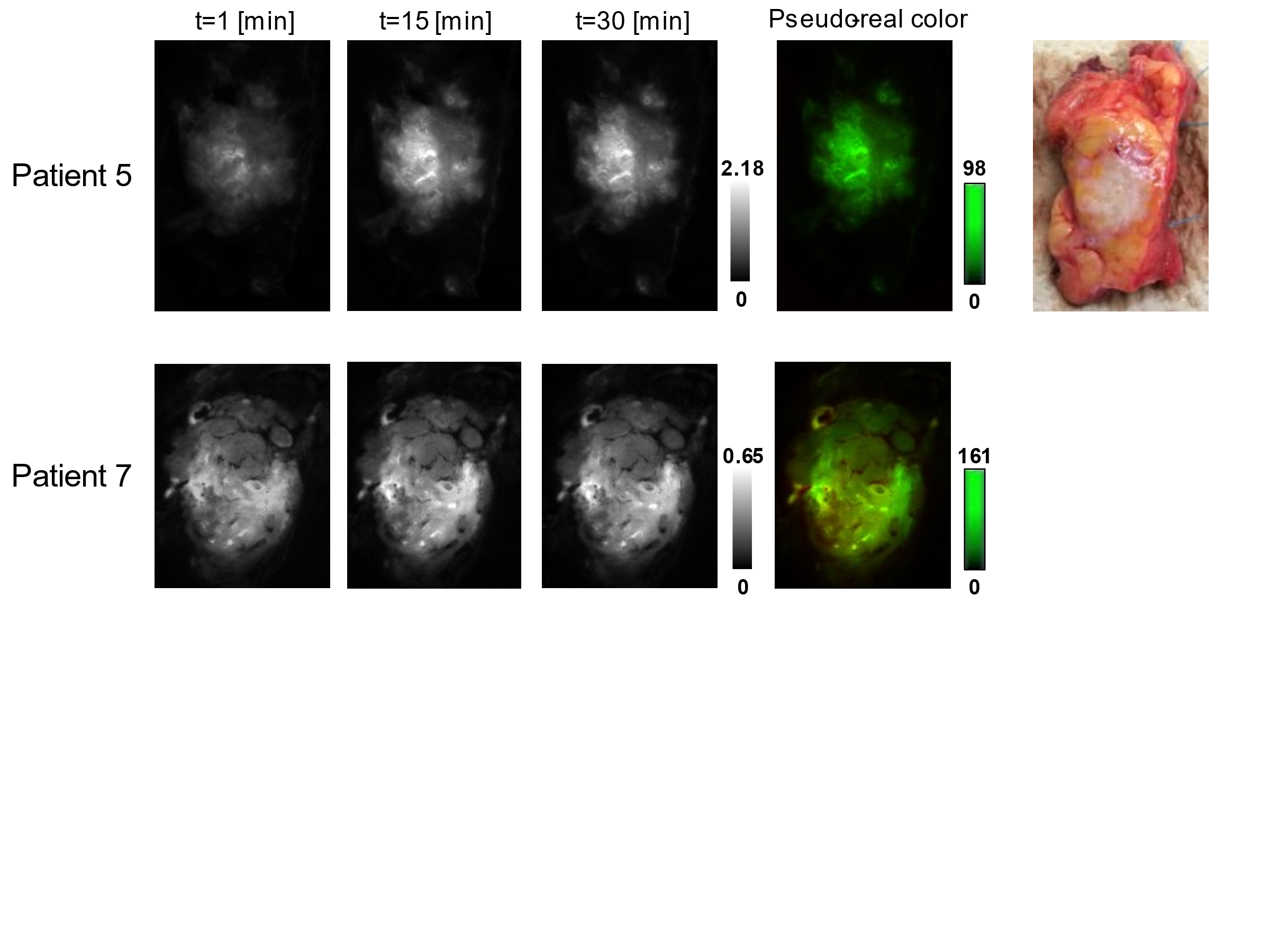

Supplement: Supplementary file 1 [file DataSheet_1.docx]
